# Temperature-Dependent Characterization of Long-Range Conduction in Conductive Protein Fibers of Cable Bacteria

**DOI:** 10.1021/acsnano.4c12186

**Published:** 2024-11-12

**Authors:** Jasper R. van der Veen, Silvia Hidalgo Martinez, Albert Wieland, Matteo De Pellegrin, Rick Verweij, Yaroslav M. Blanter, Herre S. J. van der Zant, Filip J. R. Meysman

**Affiliations:** † Department of Quantum Nanoscience, Kavli Institute of Nanoscience, Delft University of Technology, Delft 2628 CJ, The Netherlands; ‡ Department of Biotechnology, Delft University of Technology, Delft 2629 HZ, The Netherlands; § Department of Biology, Excellence Center for Microbial Systems Technology, University of Antwerp, Wilrijk 2610, Belgium

**Keywords:** cable bacteria, biological electron transport, protein fibers, conductivity, nuclear tunneling

## Abstract

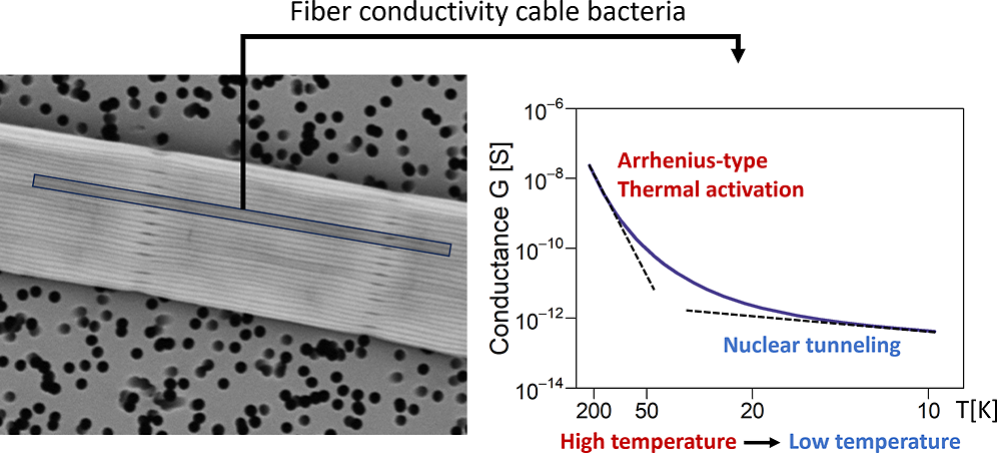

Multicellular cable bacteria display an exceptional form of biological conduction, channeling electric currents across centimeter distances through a regular network of protein fibers embedded in the cell envelope. The fiber conductivity is among the highest recorded for biomaterials, but the underlying mechanism of electron transport remains elusive. Here, we performed detailed characterization of the conductance from room temperature down to liquid helium temperature to attain insight into the mechanism of long-range conduction. A consistent behavior is seen within and across individual filaments. The conductance near room temperature reveals thermally activated behavior, yet with a low activation energy. At cryogenic temperatures, the conductance at moderate electric fields becomes virtually independent of temperature, suggesting that quantum vibrations couple to the charge transport through nuclear tunneling. Our data support an incoherent multistep hopping model within parallel conduction channels with a low activation energy and high transfer efficiency between hopping sites. This model explains the capacity of cable bacteria to transport electrons across centimeter-scale distances, thus illustrating how electric currents can be guided through extremely long supramolecular protein structures.

## Introduction

Electron flow through proteins is central to the functioning of living organisms, as it connects the sites where oxidation and reduction half-reactions take place, thus enabling a tight control of biochemical redox processes.^1^ Quantum tunneling shows an exponential dependence of the electron transfer rate on distance, which limits individual electron transfer events to distances ≤1.4 nm.^2,3^ Still, it is well-known that protein structures can support electron transport over much longer distances, as exemplified by the membrane complexes in mitochondria^4^ and chloroplasts.^5^ Biology has resolved this problem by arranging cofactors in chains at close spacings (typically 10–15 Å). These cofactors act as relay centers, so that electrons can move by through the protein medium by consecutive tunneling steps.^6^

In general, this multistep electron hopping process involves a limited number of cofactors (<20), and so the overall length scale of protein conduction remains restricted to ≤10 nm.^7,8^ Still, compelling evidence has accumulated that biological electron transport may greatly surpass this nanoscopic length scale. Some metal-reducing bacteria such as *Geobacter* and *Shewanella* mediate electron transport over micrometer distances through thin surface appendages so they can use solid electron acceptors in the external environment.^9−11^ Likewise, sizable currents can be channeled across synthetically assembled protein structures over mesoscopic ∼10–100 nm^12^ to microscopic ∼100 μm distances.^13^ These observations motivate mechanistic studies into long-range protein conductance, whose deeper understanding is not only central to biology, but also critical for the technological application of protein-based electronic materials.^14,15^

The discovery of millimeter- to centimeter-scale conduction in cable bacteria^16,17^ redefines the concept of “long-range transport” in proteins.^18^ Cable bacteria are long, multicellular bacteria that thrive in the surface sediments of rivers, lakes, and oceans.^19−21^ Their respiratory metabolism couples the oxidation of free sulfide (H_2_S) to the reduction of oxygen (O_2_), which are carried out by cells at different ends of the centimeter-long filaments.^22^ To ensure that these redox half-reactions remain electrically coupled, electrons are internally conveyed along the cable bacterium filaments.^23^ To mediate this centimeter-scale electron transport, cable bacteria harbor an internal conductive network,^24,25^ which consists of protein fibers that run in the cell envelope along the entire length of the bacterial filaments.^26−28^ Recent evidence suggests that these protein fibers harbor a sulfur-ligated nickel compound that likely acts as a cofactor in the electron transport.^28,29^ This compound shares a resemblance with nickel bis(1,2-dithiolene) complexes, which suggest that its structure markedly differs from the known Ni-cofactors in biology.^29^ Room-temperature characterization has further revealed that these protein fibers can attain a conductivity in excess of 100 S/cm,^26,28,30^ which is among the highest recorded for biomaterials, and even exceeds the conductivity of most organic semiconductors.^31^ However, the question as to how these long protein structures can efficiently sustain conduction over macroscopic distances remains largely unresolved.

To examine the long-range charge transport in cable bacteria, we extracted the fiber network from native bacteria and characterized the conductance of the periplasmic fibers over a wide temperature range from room temperature down to liquid helium temperature. Investigation of low-temperature conduction is not so much physiologically relevant, but is highly instrumental to get insight into the mechanism of electron transport, as thermal excitations become suppressed when the temperature is sufficiently decreased. As such, low-temperature characterization has been used to investigate the mechanism of electron transport in photosynthetic reaction centers,^32,33^ single amyloid crystals^34^ as well as thin protein layers sandwiched between contact electrodes.^35^ Here, our measurements reveal a marked shift in the electron transport with temperature. At elevated temperatures charges are transported through multistep hopping, involving low-energy barriers that may originate from delocalized charge carrier wave functions. At temperatures below 75 K and when applying moderate electric fields, the conductivity becomes nearly independent of temperature, consistent with the presence of quantum vibration effects through nuclear tunneling.

## Results

### Electric Characterization of Long-Range Transport in Individual Cable Bacterium Filaments

**Figure 1:**
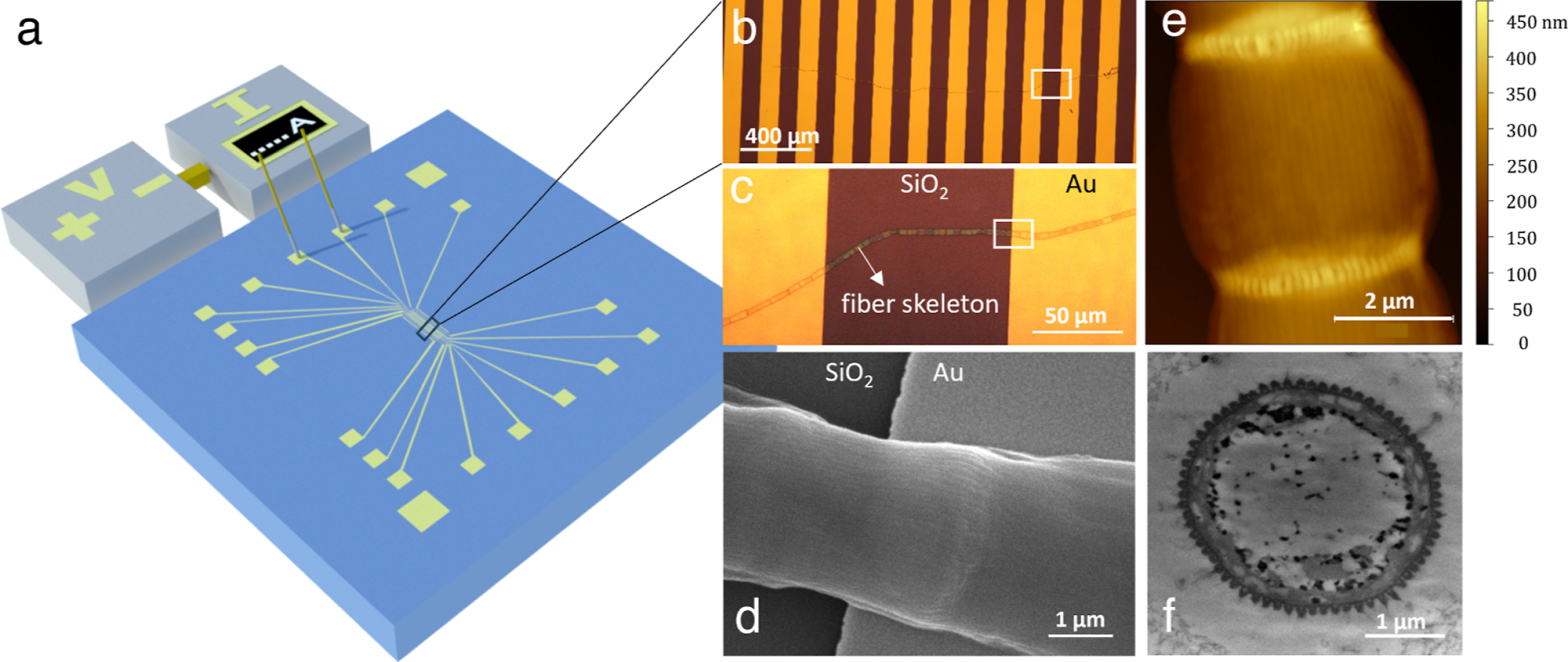
Electric characterization of fiber skeletons from cable bacteria. (a) Individual fiber skeleton filaments are deposited on Si/SiO_2_ substrate with prepatterned gold contacts. Two-probe and four-probe current/voltage measurements are conducted. (b) A fiber skeleton is stretched across a series of gold contacts. (c) A fiber skeleton segment bridges the nonconductive gap between two electrodes. Individual cells are notable that make up the filament. The rectangle delineates the zoom-in of panel b. (d) Scanning electron micrograph of a fiber skeleton on the substrate. The conductive fibers are seen as lines running in parallel along the bacterial filament. (e) Topography image of a fiber skeleton as obtained by atomic force microscopy. The light-colored rings represent the cell–cell interfaces. The color bar indicates the height. (f) Transmission electron micrograph of a cross-section of a native cable bacterium filament. The cell surface displays *N*_F_ = 68 ridges. The conductive fibers are seen as light colored patches within these surface ridges.

Electrical characterization was performed on fiber networks isolated from native cable bacteria (Figure 1). To this end, long filaments of the marine cable bacterium *Candidatus Electrothrix gigas* were individually isolated from enrichment cultures.^36^ Sequential extraction selectively removed the membranes and cytoplasm to produce a so-called “fiber skeleton” that retains the periplasmic fiber network lying on top of a connective carbohydrate skeleton.^26^ These fiber skeletons display a similar conductivity as native filaments, indicating that the applied extraction procedure does not structurally or functionally affect the conductive fiber network.^26^ Raman spectroscopy and atomic force microscopy (AFM) were used to verify the reproducibility and quality of the extraction procedure (see Methods). Fiber skeleton filaments (diameter 4 μm; length 2–4 mm) were individually placed onto silicon substrates with prepatterned gold electrodes separated by nonconductive gaps of variable size (Figure 1a–c). Microscopy revealed how single filaments stretched across multiple electrodes (Figure 1d), which allowed to compare the conductance of different segments within the same filament. Transmission electron microscopy (TEM) on cross sections was used to determine the number of conductive fibers within a fiber skeleton (*N*_F_ = 68; Figure 1f). Knowing *N*_F_ and the segment length *L*, one can calculate the mean fiber conductivity σ within from the recorded conductance *G* (see eq 5 in Methods).

**Figure 2:**
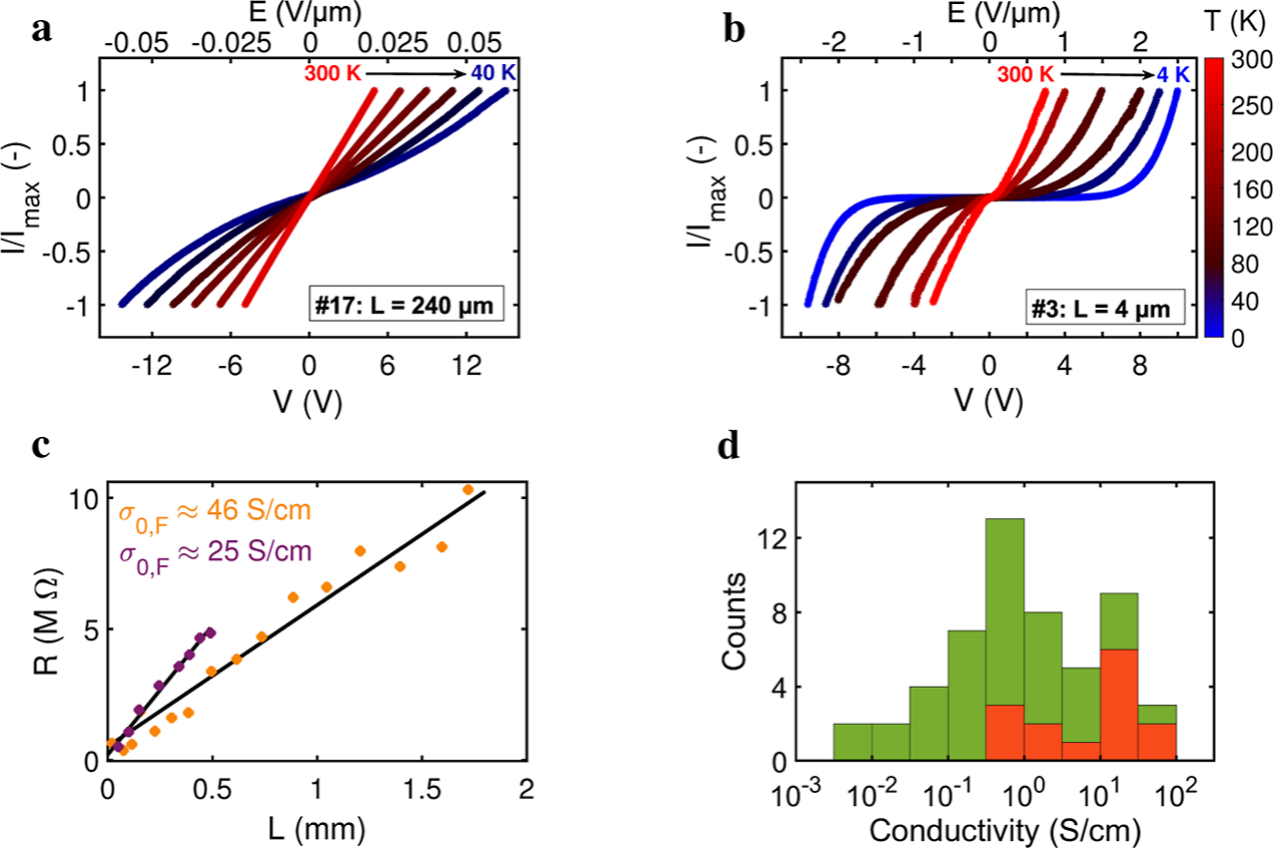
Current–voltage characteristics. (a,b) Representative two-probe measurements of the current (*I*) versus bias voltage (*V*) at different temperatures for two different segment lengths (*L* = 240 μm and *L* = 4 μm). To enable a comparison of curve shapes, the current is normalized to the maximum current of each *I*(*V*) trace. Temperature is indicated by the color scale. The top axis displays the applied electric field, *E* = *V*/*L*. Shorter segments allow to investigate the conductance at lower temperatures and higher electric fields. For the longer segment, the current fell below the detection limit below 40 K. (c) Four-probe measurements of the resistance, *R*, as a function of the electrode spacing, *L*, at 300 K for two long fiber skeletons. The lines represent linear fits through the data which provide an estimate of the fiber conductivity (inset values). (d) Histogram of the room-temperature conductivity σ_0_ for all fiber skeleton segments investigated (*n* = 53). Four-probe values (red bars; *n* = 14) reflect the intrinsic fiber conductivity and are higher than two-probe values (green bars; *n* = 39) due to the absence of the contact resistance.

Current (*I*)–voltage (*V*) characteristics were recorded for *n* = 53 segments of varying length (*L* = 4 μm to 2 mm), originating from *n* = 21 separate fiber skeleton filaments (Table S1). All segments (*n* = 53) were investigated over the temperature range 300 to 100 K, while a selection of segments (*n* = 16) was measured down to cryogenic temperatures (4.2–10 K) and characterized in more detail. Figure 2a,b displays representative *I*(*V*) data for a long (*L* = 240 μm) and short segment (*L* = 4 μm). Figure S1 provides the data for the 14 other segments that were investigated down to cryogenic temperatures. Measurements were highly consistent between segments, revealing similar *I*(*V*) characteristics (Figure S1). The measured current shows a distinct response to the temperature, *T*, and to the imposed electric field, *E* = *V*/*L*. At high temperature and low electric field, the *I*(*V*) curve is linear (Figure 2a). Yet, when temperature decreases and the electric field strength increases, the *I*(*V*) becomes increasingly nonlinear (Figure 2a,b). *I*(*V*) curves remained symmetric for positive and negative bias, and did not change upon repeated voltage sweeps.

When performing low-temperature electric characterization of protein structures, one should be vigilant for artifacts. To assess the impact of contact resistances,^26,34^ two- and four-probe measurements were done on the same segment. The contact resistance varied between 12% and 79% of the total resistance. While the fiber conductivity is higher in a four-probe configuration (Figure S3), the activation energy is similar for two-probe and four-probe recordings (Figure S3), and so contact resistances do not appear to influence the temperature dependence of the conductance. For some segments, the four-probe current dropped below detection limit at the lowest temperatures, and as a result, our two-probe data set is more extensive. The maximal bias voltage imposed upon a segment scaled with the segment length, so that the maximal electric fields imposed (*E* < 2.5 V/μm) were comparable those typically used for the investigation of protein junctions^35^ and bacterial nanowires.^37^ This avoids breakthrough currents and Joule heating incurred by high electric fields. The estimated temperature increase upon current passage (Δ*T* < 0.06 at 10 K) remains indeed small across all segments and conditions investigated (Table S3). The gold electrode contacts were large (100 μm), thus spreading the current injection over a large zone of fiber skeleton, and hence reducing the risk of heating at the contacts. The fact that the *I*/*V* remained invariant upon repeated voltage sweeps suggests that heating within the fibers or contacts—if present—does not have a marked influence on the *I*/*V* curve. Furthermore, the activation energy (as discussed below) showed no correlation with conductivity (Figure S3b), which also argues against a bias induced by Joule heating.

### Room Temperature Resistance Increases Linearly with Segment Length

For all segments investigated, the differential conductance *G*_0_ at room temperature was determined from the slope of the *I*(*V*) curve at zero bias, and the associated mean fiber conductivity σ_0_ was calculated (Methods; Table S1). As expected, four-probe conductivities (mean: 18.4 S/cm; *n* = 14) were consistently higher than two-probe values (mean: 3.3 S/cm, *n* = 39), due to the absence of the contact resistance (Figure 2d). The four-probe data reflect the intrinsic fiber conductivity, of which the magnitude and range are consistent with prior room temperature assessments.^26,30^ The maximum four-probe conductivity obtained (74 S/cm) is high for a biological material, being 3 orders of magnitude higher than the intrinsic conductivity of *Geobacter* OmcS and OmcZ nanowires at room temperature and physiological pH^11,37^ and on par with that of highly doped synthetic organic polymers.^38^ It should be noted that these values are recorded in dry state and at high vacuum, which is far from the conditions within living bacteria. However, it has been recently shown that the fiber conductivity measured in high vacuum is similar to that in electrolyte solutions mimicking physiological conditions.^39^ This suggests that the σ_0_ values recorded are relevant for the in vivo operation.

The multiple contacts per filament allow the resistance to be evaluated on different segments of the same fiber skeleton (Figure 1b). The four-probe technique was applied such that the two current-carrying electrodes were positioned at the terminal contacts of a filament, while one voltage-sensing electrode was kept at a fixed position and the second voltage-sensing electrode was varied along the filament. The intrinsic resistance *R* showed a linear Ohmic dependence on the probed segment length *L* over a distance of millimeters (Figure 2c). A linear fit yielded σ_0_ values (25 and 46 S/cm) consistent with the four-probe fiber conductivities obtained from *I*(*V*) curves (Figure 2d). This observed linear length dependence indicates that the conductive fiber network displays homogeneous electric properties along the entire filament. This suggests that number of conduction channels as well as the charge carrier density in these channels remain constant over distances from a few micrometers up to several millimeters (Figure 2c).

Our data indicate that σ_0_ is constant within one filament (Figure 2c), but that there is considerable variation between filaments (Figure 2d). This variation has been seen before in room-temperature assessments of the conductivity,^26,39^ and hence, it is not a particular feature of the measurement approach adopted here. Presently, it is not understood whether this represents a true biological variation between filaments, or whether this arises from the filament isolation procedure.^26^ The filament handling procedure may incur physical damage on the fiber network, while also imposing variable amounts of oxygen exposure, which is known to diminish conductivity.^26^ Likewise, the conductive fiber network forms a complex biological structure, which features many parallel conduction channels as well as lateral connections between fibers. This network complexity provides many different potential current paths between charge injection and ejection sites. Not all of these pathways may be functional or connected, thus inducing variability in the overall conductance. Finally, the two-probe conductivity range is twice as wide as the four-probe range (Figure 2d), thus suggesting an impact of variable contact resistances. Yet overall, segments with a different fiber conductivity show a similar response toward temperature and electric field (Figure S3b; see also discussion below), thus indicating that charge transport mechanism is similar across the filaments investigated.

### Multistep Hopping Transport at Higher Temperatures

**Figure 3:**
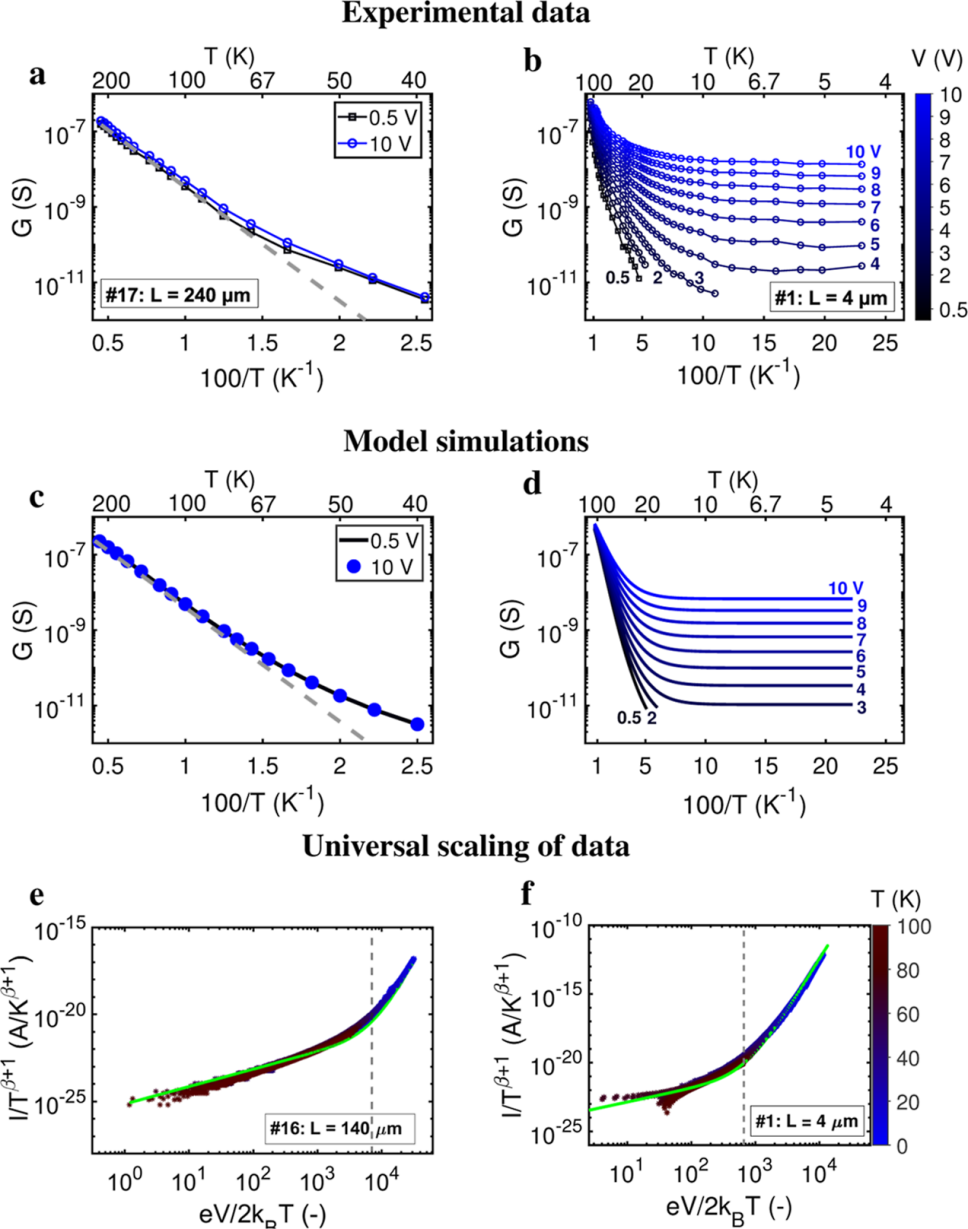
Dependence of conductance on temperature and electric field. (a,b) The nondifferential conductance *G* = *I*/*V* is plotted as a function of inverse temperature for two segments of different length. Curves are drawn at a fixed electric field strength (i.e., fixed bias voltage). In panel (a), the Arrhenius fit to the high-temperature conductance data is included for reference (dashed gray line). (c,d) Model simulation of the conductance data in panels (a,b) via a 1D hopping chain model with one effective vibrational mode (Jortner model). Model parameters in panel (c): reorganization energy, λ = 0.35 eV and effective vibrational frequency, ⟨ω⟩ = 122 cm^–1^ (15 meV). The number of hopping sites, *N*_S_, does not change the shape of the curve. Model parameters in panel (d): ⟨ω⟩ = 58 cm^–1^ (7.2 meV), λ = 0.16 eV, number of hopping sites, *N*_S_ = 200. (e,f) *I*(*V*,*T*) data replotted as a universal scaling curve;^40,41^*T* is temperature, *e* is the elementary charge, and *k*_B_ is the Boltzmann’s constant. The green solid line represents a model fit to the data with two fitting parameters: the dimensionless exponent β and the number of hopping sites, *N*_S_. For the longer segment, *N*_S_ = 1400 and β = 6.0; for the shorter segment, *N*_S_ = 120 and β = 6.5.

To attain additional insight into the electron transport mechanism, we performed a more detailed analysis on a subset of segments (*n* = 16) that were measured down to cryogenic temperatures (Table S2). When replotting the *I*(*V*) data in terms of the nondifferential conductance, *G* = *I*/*V*, we consistently observe the same *G*(*T*,*E*) response across all segments, irrespective of σ_0_ (Figure 3a,b displays data for a short and long segment; Figure S1 shows all other segments). Two distinct temperature regimes are apparent, marked by a crossover temperature *T*_C_ ∼ 75 K. At high temperatures (*T* > *T*_C_), charge transport is thermally activated (Figure 3a) and follows an exponential Arrhenius-type dependence.^30^ Within this high-temperature regime, the *G*(*T*) curves collected for different *E*-values converge (Figure 3a), which implies that the conductance is not affected by the electric field. Below *T*_C_, the temperature dependence becomes noticeably weaker and the conductance is strongly affected by the electric field (Figure 3b). For example, at 5 K, *G* increases by 3 orders of magnitude when *E* is increased from 1 to 2.5 V/μm (Figure 3b). At these low temperatures (*T* < 20 K), the conductance becomes virtually independent of temperature for the higher electric fields applied.

While coherent electron transport has been observed in protein junctions over nanometer distances,^35,42^ longer range conduction in proteins is conventionally described by a multistep hopping formalism, which assumes that charges are temporarily localized at a particular site and electron transport occurs by incoherent “hopping” between consecutive sites along a chain.^8,43^ To verify whether multistep hopping can explain the observed *G*(*T*,*E*) response, we developed a transport model that describes the conductive fiber network in cable bacteria as a set of parallel one-dimensional hopping chains (Figure S5; model specification in Supporting Information based on ref (44)). Each hopping chain has *N*_S_ = *L*/δ charge carrier sites, where δ presents the center-to-center distance between two consecutive sites. Similar hopping models have been used to describe electron transport in multiheme cytochromes,^45^ protein-based molecular junctions,^46^ and bacterial nanowires.^43,47,48^

The *G*(*T*,*E*) response predicted by the model is critically dependent on the kinetic expression implemented for the transition rate Γ during a single hopping step. If we describe this electron transfer by standard nonadiabatic theory, the forward transition rate Γ_F_ is given by classical Marcus expression^49^
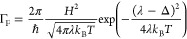
1here, *H* represents the electronic coupling between the initial and final states, λ is the reorganization energy, and ℏ and *k*_B_ denote the reduced Planck and Boltzmann constants, respectively. When a voltage bias *V* is imposed across the terminal electrodes, it is distributed equally over all the sites within the hopping chain, thereby providing a driving force Δ = *eV*/*N*_S_ = *eE*δ for the charge transport between two consecutive sites. When the driving force remains small compared to the reorganization energy (Δ < λ), the conductance of the fiber network scales as (Supporting Information)
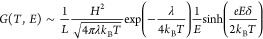
2

As noted above, the electric field *E* stays small for the long segments investigated here, which hence provides values of Δ smaller than *k*_B_*T* = 25 meV at room temperature (see additional discussion in Supporting Information). So within the high-temperature regime, the driving force remains small compared to the thermal energy scale (Δ ≪ *k*_B_*T*). As a result, the approximation sinh(*x*) ≈ *x* holds in eq 2, and the conductance relation becomes independent of *E* (Supporting Information).
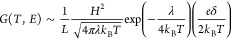
3

**Figure 4:**
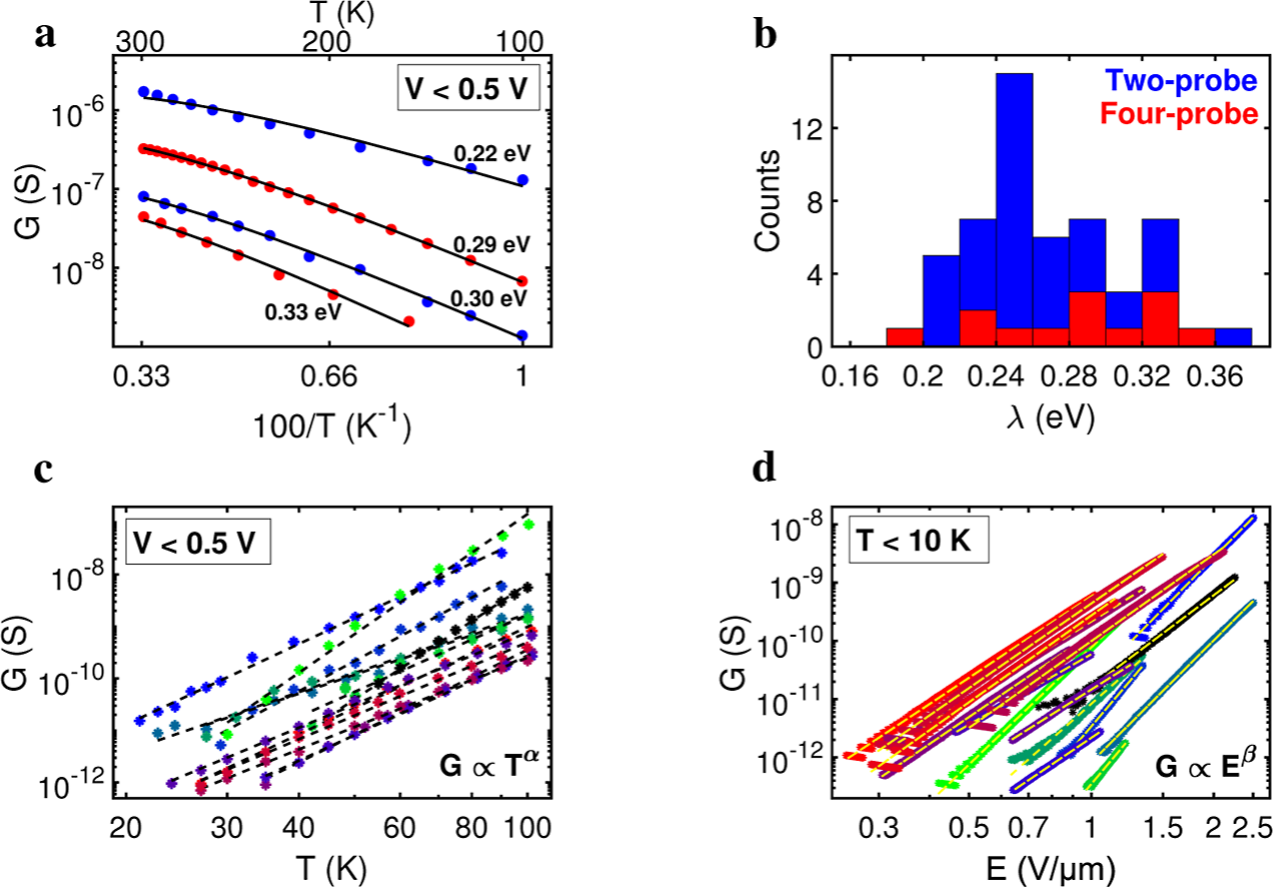
Temperature dependence of the conductance. (a) Low-bias conductance, *G*, as a function of inverse temperature for *T* > 100 K. Data for 4 representative segments. Two- and four-probe measurements are shown in blue and red markers, respectively. Solid black lines represent model fits by eq 3. (b) Histogram with reorganization energies for *n* = 53 samples (blue: two-probe measurements; red: four-probe measurements). (c) Low-bias conductance plotted against temperature in the region 20–100 K for 16 segments. Black dashed lines represent power law fits (determining α). (d) Conductance versus applied electric field at the lowest temperature (*T* < 10 K), for the same 16 segments. Yellow dashed lines represent power law fits (determining β).

This explains why the *I*(*V*) curves are linear for the larger segments (Figure 2a), why the resistance linearly scales with the chain length (Figure 2c), and also why the conductance remains independent of *E* at higher temperatures (Figure 3a). Conversely, for lower temperatures and shorter segments, the *I*(*V*) curve is predicted to adopt a nonlinear, hyperbolic sine shape, as also seen in the data (Figure 2b). The observed temperature dependence of *G* provides further support to the multistep hopping model. The conductance follows the *G* ∼ *T*^–3/2^ exp(−λ/4*k*_B_*T*) dependence predicted by eq 3 for *T* > 100 K (Figure 4a). The reorganization energy is low, λ = 270 ± 40 meV (*n* = 53 segments; Table S1), and reveals no significant difference between two-probe and four-probe measurements (Figure 4b). The corresponding activation energy, *U*_A_ = 41 ± 8 meV (*n* = 53; Table S1), as obtained by fitting *G* ∼ exp(−*U*_A_/*k*_B_*T*) (see Figure S4), aligns well with previous high-temperature measurements on both intact cable bacteria and fiber skeletons.^30^ Both λ and *U*_A_ show limited variation between segments and display no dependency on σ_0_ (Figure S3). Accordingly, the low reorganization and activation energy appears to be a robust feature within the high-temperature regime, which also includes the range of biologically relevant temperatures.

### Quantum Vibrations Couple to Electron Transport at Low Temperatures

While classical Marcus theory explains the observed *G*(*T*,*E*) response at higher temperatures, the conductance deviates from the expected Arrhenius behavior below the crossover temperature *T*_C_. The conductance remains elevated at the lowest temperatures and increases with the application of an electric field (Figure 3b). This suggests an energy source other than thermal energy that assists the charge transfer. In effect, our data closely resemble those recorded by DeVault and Chance in their seminal experiments in the 1960s on photosynthetic reaction centers in purple bacteria. These experiments revealed that in the charge separation reaction of photosynthesis, the reaction rate eventually becomes temperature-independent at cryogenic temperatures.^32,33^ At the time, these findings could not be explained by classical Marcus theory, and so the DeVault and Chance experiments spurred a host of theoretical developments. This extended electron transfer theory to a fully quantum-mechanical treatment that explicitly accounts for the impact of quantum vibrations^40,50−52^ that retain a finite zero-point energy at absolute zero. At low temperatures, thermal motions are frozen out, but quantized vibrational modes can still assist the electron tunnelling, in a process referred to as nuclear tunneling.^50,51^

To verify whether nuclear tunnelling could explain our observations, we adapted our multistepping transport model and replaced the Marcus rate eq 1 by transition rate expressions that account for quantum vibrational coupling (Supporting Information Text). In protein structures, vibrational coupling can involve both inner-sphere modes, i.e., high-frequency intramolecular vibrations of the charge carrying cofactors, as well as outer-sphere modes, i.e., lower-frequency vibrations of the surrounding protein matrix and solvent molecules. This vibrational coupling can be described with different models of increasing complexity. As a first step, we applied a simplified model where only one effective high-frequency mode ⟨ω⟩ is assumed to stimulate the hopping process (Jortner model,^51^Supporting Information Text). This model suitably captures the main feature of our experimental data: the conductance displays two regimes with a transition at the crossover temperature *T*_C_ = ℏ⟨ω⟩/*k*_B_. For *T* > *T*_C_, the vibrational mode remains thermally excited, and the conductance adopts a classical Arrhenius-type dependence as predicted by classical Marcus theory (Figure 3c). Below *T*_C_, the vibrational mode is no longer thermally excited, but due to the quantum vibrational energy, the conductance remains higher than predicted by the Arrhenius relation. Within this low-temperature regime, the conductance also displays a marked dependence on the electric field, as observed in the data (Figure 3d).

The Jortner model condenses all vibrational modes into one effective mode, which comprises a strong simplification. In biomolecular structures like proteins, multiple modes will couple to the electron transport at a given temperature. Closer data inspection indeed suggests that a combination of both classical and quantum modes is likely involved. Rather than becoming constant (as predicted by the Jortner model), the conductance at low bias continues to decrease as a power law (*G* ∝ *T*^α^; Figure 4c). To account for this, we integrated a multimode description of vibrational coupling in our multistep hopping model, which adopts an Ohmic spectral density with an upper cutoff frequency to describe the coupling between the charge and the nuclear bath (Egger model,^40^Supporting Information Text).

There are several predictions that can be tested by the Egger model. Foremost, this multimode model predicts that the conductance should follow a power-law dependence on temperature at low electric fields, *G* ∝ *T*^α^, and a power-law dependence on the electric field at low temperatures, *G* ∝ *E*^β^.^41^ The conductance indeed shows such a power law behavior with exponents α ≈ 3.7–7.7 and β ≈ 4.0–8.3 (Figure 4c,d; Table S2). The large value of β exemplifies the strong field dependence of the conductance at low temperatures and moderate fields (Figure 3b). Moreover, the multimode model asserts that in the low-temperature regime, the *I*(*V*,*T*) data for a given segment can be suitably reduced to a single universal scaling curve^41^ (see also Supporting Information Text), which features the normalized current *I*/*T*^1+β^ as a function of a normalized bias .
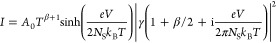
4

Our *I*(*V*) data follow this universal scaling curve for all 16 segments investigated (Figure 3e,f; Figure S2). Interestingly, this universal scaling behavior is also characteristic for various synthetic, carbon-based one-dimensional conductors, such as networks of graphene nanoribbons^53^ and carbonized polymer nanofibers,^54^ which display similarly high conductivities as the periplasmic fibers in cable bacteria.

## Discussion

The data presented here provide several insights into the underlying mechanism of long-range conduction in the fiber network of cable bacteria. First of all, the charge transport mechanism shows a marked shift with temperature. At high temperatures, electron transport is assisted by thermal fluctuations, thus providing an Arrhenius dependence, while at low temperatures, molecular vibrations couple to the electron transfer, thus resulting in higher conductance than classically expected (Figure 3). Overall, the observed temperature dependence is consistent with a multistep hopping mechanism, where the driving force changes from thermal activation toward nuclear tunneling as the sample is cooled down below the crossover temperature *T*_C_. While the appearance of quantum features in the electron transfer rate at low temperature is well-known,^40,52,55^ there are several features notable about the mechanism of long-range charge transport in cable bacteria.

Foremost, the multistep hopping in cable bacteria takes place over distances from millimeters to centimeters. If cofactors feature as relay sites for the electron transport, these cofactors must then be spatially aligned over the same macroscale distances. Raman spectroscopy has shown that the periplasmic fiber network does not contain FeS clusters nor cytochromes.^26,28^ This excludes a heme-based conduction mechanism as found in the surface appendages of metal-reducing bacteria.^11,48,56^ Instead, recent work suggests that the periplasmic fibers in cable bacteria contain a nickel–sulfur cofactor that mediates the electron transport.^28,29^ Therefore, the vibrations that couple to the electron transport, thus giving rise to nuclear tunneling, are most likely connected to this nickel–sulfur cofactor. The involvement of nickel is noteworthy, as currently known metalloproteins involved in electron transport rely on either iron- or copper-containing cofactors, but not on nickel.^57^

A second notable feature of the current data is that the reorganization energy at room temperature (0.27 eV) is markedly low. Values for single-electron transfer steps in enzymes are in the range of 0.7 < λ < 1.4 eV, and multistep electron transport in the surface appendages of the metal-reducing bacteria exhibits a reorganization energy of 0.8–1.0 eV.^58,59^ One exception appears to be the blue copper protein azurin, which shows almost activationless transport when a monolayer is sandwiched between electrodes in a solid-state configuration.^60^ There are multiple factors that can lower the reorganization energy. Electron transfer reactions that employ fewer vibrational modes tend to have a lower reorganization energy, while changes in polarizability of the active sites or nonergodic dynamics originating from the protein scaffold can also give rise to lower reorganization energies.^61^ Furthermore, the reorganization energy is also known to inversely scale with the localization length of the charge carriers,^62^ and so, the low reorganization energies seen here could suggest delocalization of the charge carrier wave function across relatively large distances (nanometers). Comparably low reorganization energies have been reported for highly mobility organic semiconductors, such as rubrene and naphtalene,^63^ in which the charge carrier sites also display sizable conjugation and delocalization. Finally, near room temperature, the hopping rates may increase up to a point that they become comparable to the mode frequencies. In the diabatic regime, when the electron transfer rates are higher than the mode frequencies, the fluctuation dissipation theorem no longer holds and this could entail a further reduction of reorganization energy.^61^ At present, we have insufficient knowledge about the system parameters to quantitatively determine where the nonadiabatic regime ends, but the observation that the Arrhenius dependence still holds for higher temperatures however suggests that the electron transport may still stay within nonadiabatic regime.

A final, important consideration concerns the hopping frequency Γ and center-to-center spacing δ in the hopping chain. In our multistep hopping model, these quantities are related via Γ = σ(4*k*_B_*TA*_F_)/(*e*^2^*N*_C_δ), where *N*_C_ refers to the number of parallel conduction channels in a single fiber. At present, the values of *N*_C_ and δ are unknown, but we can still impose constraints. It has recently been suggested that a single periplasmic fiber of the cable bacteria consists of a bundle of interwoven fibrils (diameter 4 nm), and as such, one fiber could accommodate up to *N*_C_ = 30 separate parallel conduction channels. The center-to-center spacing of charge carrier sites in these fibrils is presently unknown. But if we assume that these fibrils would have a similar cofactor packing as the hemes in the OmcS nanowires of *Geobacter* (δ = 0.78 nm;^37^), then for a fiber conductivity σ_0_ = 18 S/cm (mean value of four-probe data), the required transition frequency becomes 2.7 × 10^13^ s^–1^ (see details in Supporting Information). This <0.1 ps electron transfer rate is orders of magnitude faster than currently observed for nonlight driven electron transfer, which is typically on the scale of microseconds in membrane-bound multiheme cytochromes^8^ and reaches 0.1 ns in the OmcS nanowires of *Geobacter*.^37^ Effectively, this 10^13^ s^–1^ hopping frequency exceeds the speed limit of nonadiabatic electron transfer, where relaxation times of vibrational modes are slower than the hopping rate itself.^47,64^ As such, the high conductivity recorded in cable bacteria poses a clear challenge to a classical, nonadiabatic multistep hopping model.^44^

One way to resolve this is to adopt a larger center-to-center distance δ, while retaining the same edge-to-edge distance between cofactors. This enlargement of the cofactor allows for a smaller hopping rate for the same conductivity, thus bringing the electron transport back in the nonadiatabic regime.^43,44^ Our data enable two separate estimates for the number of hopping sites *N*_S_ within a segment of length *L*; one estimate is directly inferred from the universal scaling curve, the other is based on *G*(*T*,*E*) model fitting (Supporting Information). Both methods provide similar *N*_S_ values, which linearly scale with the segment length *L* (Figure S6), thus providing a center-to-center distance between hopping sites in excess of 10 nm (Table S1). Clearly, this distance is far too large to enable through-space tunneling of electrons,^65,66^ and largely exceeds the known heme-to-heme distances in cytochromes (<1 nm).^45,67^ In doped organic semiconducting nanowires, like polyacetylene, the universal scaling curve provides similar center-to-center distances (∼10 nm).^41,54,68^ These longer length scales have been linked to energy correlations associated with charge–dipole interactions that extend over more than 10 hopping sites.^41^

An extended version of Marcus theory allows charge transfer between so-called donor and acceptor “aggregates”, in which the charge is no longer localized on a single cofactor, but delocalized across a cluster of multiple cofactor molecules.^62^ In this view, the electronic wave function is spread over a “block” of cofactors, and the electron transport comprises a combination of coherent transport within blocks and incoherent hopping between blocks.^44^ Such mixed hopping-coherent models have also been proposed for the nanowires in*Geobacter*,^43,69^ in which the coherent part is thought to result from a local rigidification of the protein structure.^43^ Note that if hopping takes place between such “cofactor blocks”, it would imply that the length scaling obtained from the universal scaling curve should be no longer interpreted as a center-to-center distance between individual cofactors.

## Conclusions

In conclusion, this study details how the conductance in the periplasmic fibers of cable bacteria varies from room temperature down to liquid helium temperature. A consistent behavior is seen within and across individual filaments. At higher temperatures, thermally activated behavior provides an Arrhenius dependence, while at cryogenic temperatures, the conductance becomes virtually independent of temperature, suggesting nuclear tunneling. These data provide a critical resource to better understand the long-range charge transport in these biological wires. In future experiments, the observed conductance behavior should be linked to the molecular nature and arrangement of the tentative nickel cofactors^28^ that are embedded within the periplasmic fibers. This way, a structural basis can be established for the high conductivity recorded in cable bacteria. Ultimately, this may enable the design and construction of conductive biomimetic materials for electronics and energy conversion.

## Methods

### Extraction of Conductive Fiber Networks from Cable Bacteria

Cable bacterium filaments were harvested from enrichment cultures with natural sediment collected in the creek bed of a saltmarsh (Rattekaai, The Netherlands). Upon collection, sediment was sieved and repacked into PVC core liner tubes (diameter 40 mm). The cores were incubated in aerated artificial seawater at in situ salinity, and the development of cable bacteria was tracked by microsensor profiling and microscopy, following the procedure as in ref (19). Under a stereo microscope, individual filaments were gently pulled out from the top layer of the sediment with custom-made glass hooks. To remove debris and attached sediment particles, filaments were cleaned by transferring them at least six times between droplets (∼20 μL) of Milli-Q water on a microscope coverslip. Based on size and morphology (as determined via SEM, TEM and AFM microscopy), cable bacterium filaments were identified as *Ca. Electrothrix gigas*.^36^

Through sequential extraction, the conductive fiber network was isolated from the cell envelope of individual filaments.^24^ This extraction removes the membranes and cytoplasm, but retains the parallel conductive fibers embedded in a basal sheath.^28^ These so-called “fiber skeletons” form the starting material for all investigations performed here. To produce these fiber skeletons, freshly isolated cable bacterium filaments were cleaned by transferring them at least six times between droplets (∼20 μL) of Milli-Q water on a microscope coverslip. Subsequently, filaments were extracted in a droplet of 1% (w/w) sodium dodecyl sulfate (SDS) for 10 min, followed by six Milli-Q washes. Filaments were then incubated for 10 min in a droplet of 1 mM sodium ethylene diamine tetra-acetate (EDTA), pH 8, and again six times washed in Milli-Q. The extraction procedure is described in detail in refs (24 and 28).

The quality of the extraction procedure was verified by resonance Raman microscopy and atomic force microscopy. Suitably extracted fiber skeletons display the characteristic Raman fingerprint of the Ni-cofactor, but do not show a signal of cytochromes, which are removed during the SDS–EDTA extraction.^29^ Likewise, extraction the height of filaments by removing membranes as well as cytoplasmic and periplasmic material. AFM imaging was used to verify the height of the fiber skeletons, which should fall in the ≈250–350 nm range after successful extraction.

### Microscopy

To perform scanning electron microscopy (SEM), native cable bacterium filaments were dried and gold coated (Agar Sputter Coater). SEM images were obtained with a Phenom ProX scanning electron microscope (Phenom-World B.V., The Netherlands) using a backscattered electron detector at an acceleration voltage of 10 kV. For transmission electron microscopy, native cable bacterium filaments were agarose embedded in Unicryl resin and 50 nm thick sections were prepared by ultramicrotomy. Sections were stained with 2% uranyl acetate and lead citrate for 1 min, washed, and dried before being examined with a FEI Tecnai G2 Spirit BioTWIN operating at 120 kV. For atomic force microscopy (AFM), fiber skeletons were transferred onto a 50 nm gold coated silicon wafer (Platypus Technologies) in a drop of mQ water and subsequently air-dried. The wafer was securely affixed with double sided carbon tape to a 12 mm diameter stainless steel metal disc. AFM images were acquired in tapping mode with a XE-100 scanning probe microscope (Park Systems). The AFM system was equipped with an aluminum SPM probe with a tip radius <10 nm (AppNano ACTA-200), resonant frequency of 200–400 kHz, and a nominal spring constant of 13–77 N/m. Topographic and amplitude AFM data were recorded and processed with the Gwyddion software.

### Temperature-Dependent Electrical Characterization

Gold electrode patterns were deposited onto p^2+^-doped silicon substrates with a surface layer of silicon dioxide (285 or 500 nm thickness) via optical lithography. A laser writer illuminates the desired pattern in a single light-sensitive resist layer (AZ ECI 3007 or 3012). The laser wavelength (365 nm) limits the minimum feature size to approximately 1 μm. The gold thickness was 100 nm, with 5 nm of titanium underneath to promote adhesion of the gold layer to the SiO_2_ surface. Fiber skeletons were positioned onto patterned substrates immediately after extraction (Figure 1) and substrates were directly transferred to the vacuum chamber of the probe station. As known from prior studies,^26^ the conductance gradually decreases upon exposure to ambient air, and to avoid this, all electric characterization was performed under high-vacuum conditions (<10^–6^ bar). In this way, fiber skeletons retain a stable conductance for up to a period of weeks to months.^26^

The conductance of fiber skeletons was measured down to liquid helium temperatures in two separate set-ups. The first setup is based around a dewar of helium, in which a stick containing a vacuum sample chamber can be inserted. In this setup, the patterned substrates were glued to a chip carrier, either with silver paint or epoxy glue. The electrode pads of the substrate were subsequently wire-bonded to the electrodes of the chip carrier. For the lowest temperatures (*T* < 20 K) the sample chamber is not completely vacuum, because helium gas exchange is used to reach the desired temperature. The minimum temperature that can be reached approaches the helium condensation point (4.2 K). For higher temperatures (*T* > 20 K), the helium exchange gas was pumped out of the sample chamber. A resistor was used to heat up the device, while a thermometer is placed nearby the sample to measure the temperature. The second setup used was a cryo-free LakeShore Cryogenic Probe Station (Type CRX 6.5 K). In this case, the electrode pads did not need to be wire bonded. Of the 16 segments for which the temperature dependence was measured down to the lowest temperatures (*T* < 10 K; Figure S1), segments 1–5 were measured in the first setup and segments 6–16 were measured in the second.

At each temperature, multiple consecutive *I*(*V*) curves were collected to verify that the conductance signal remained stable through time. *I*(*V*) collection included both a forward and backward sweep. The measurement started at zero voltage, was run to the maximum positive voltage, then down to the (same) maximum negative value and back to zero again. We employed a sufficiently slow sweep rate as to avoid capacitance effects, and so there was no difference between the forward and backward sweep.

### Two-Probe and Four-Probe Measurements

To assess the impact of contact resistances, two-probe and 4-probe measurements were conducted. In a two-probe configuration, the measured resistance accounts for the intrinsic resistance of the probed filament segment as well as the contact resistance between the gold electrodes and filaments. A bias voltage, *V*_B_, was applied across two electrodes, and the induced current, *I*_M_, was measured. This yielded the two-probe resistance, *R*_2P_ = *V*_B_/*I*_M_ = *R*_i_ + *R*_C_, which is composed the intrinsic resistance of the fiber skeleton segment, *R*_i_, and the two contact resistances between the fiber skeleton and the electrodes, *R*_C_. In a four-electrode setup, one eliminates the influence of contact resistances and electrode polarization. In our four-probe approach, the bias current, *I*_B_, was injected over the two outer electrodes, while the voltage, *V*_M_, was measured over the two inner electrodes. The four-probe resistance, *R*_4P_ = *V*_M_/*I*_B_, equals the intrinsic resistance of the conductive fiber skeleton segment between the two inner pads. The contact resistance is hence determined as *R*_C_ = *R*_2P_ – *R*_4P_.

It should be noted that the electric field, when defined as the voltage divided by fiber length *E*_C_ = *V*/*L*, only reflects the intrinsic electric field within the fiber when there is no contact resistance, i.e., when *V* is measured in the four-probe configuration. In the two-probe configuration, part of the applied voltage will be dropped across the contacts, and so in this case, the calculated value of *E*_C_ = *V*/*L* should be seen as an indicative (maximal) value for the intrinsic electric field within the fiber.

### Filament Conductance and Fiber Conductivity

Both the nondifferential conductance (*G* = *I*/*V*) and the differential conductance (*G* = d*I*/d*V*) were calculated from the *I*(*V*) curves. The nondifferential conductance *G*(*V*,*T*), at a particular voltage *V* and temperature *T*, was calculated as the difference between the current at the positive and negative voltage divided by the voltage interval, *G* = *I*(+*V*) – *I*(−*V*)/(2*V*). This procedure takes advantage of the current–voltage characteristics being symmetric and enables the direct transformation of the *I*(*V*,*T*) curves into *G*(*E*,*T*) curves. Note that when the *I*/*V* is nonlinear, the nondifferential conductance retains a dependence on the imposed voltage bias (or equally the electric field).

Alternatively, the differential conductance *G*_0_ was determined from the slope of the *I*(*V*) curve at zero bias through linear regression, and was *G*_0_ at room temperature (*T* = 300 K). When the *I*/*V* is linear, the differential and nondifferential conductance are the same. The associated fiber conductivity, σ_0,F_, was calculated as
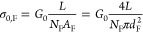
5

In this expression, *L* is the length of the fiber skeleton segment investigated as determined by microscopy, *A*_F_ is the cross-sectional area of one fiber, and *N*_F_ is the total number of fibers embedded in the fiber skeleton. The resulting values for *G*_0_ and σ_0_ are given for all segments in Table S1. The number of parallel fibers *N*_F_ was determined by TEM on cross sections of native cable bacterium filaments^24^ and was *N*_F_ = 68 (see Figure 1f). The diameter of the conductive core of an individual fiber was set to *d*_F_ = 26 nm, as previously determined by scanning dielectric microscopy.^28^

### Transfer Length Measurement

The resistance was determined as a function of the segment length. To this end, long (0.5–2 mm) fiber skeletons were individually positioned an array of electrode contacts (perpendicular to the line contacts). The intrinsic resistance *R* of the probed segment with length *L* was determined by the four-probe method. In these measurements, the two current-carrying electrodes and one voltage-sensing electrode were kept at a fixed position, while the second voltage-sensing electrode was varied along the filament, for increasing *L*. The resistance at each point was calculated as *R* = *V*/*I*, where *V* is the voltage measured across the voltage-sensing electrodes and *I* is the applied sweeping current. The fiber conductivity was derived as σ_0,F_ = 4/(*N*_F_π*d*_F_^2^*b*), where *b* is the fitted slope, and the other parameters are the same as in eq 5. Note that this approach assumes that current within the filaments is not shortened across the intervening electrodes because of the contact resistance. Yet, even when such shortening would take place, the total length of the nonconductive regions scales linearly with the total length *L*.

### Assessment of Joule Heating

The overall heating upon the passage of an electric current through a fiber skeleton can be decomposed as Δ*T* = Δ*T*_f_ + Δ*T*_s_, where Δ*T*_f_ represents the temperature increase within the fiber skeleton and Δ*T*_s_ is the localized temperature increase of the SiO_2_ underlying substrate (Δ*T* relative to the mean temperature of the probe station chamber). As the thermal conductivity of the substrate (*k* = 1.2 W/mK) is considerable higher than that of fiber skeleton protein material (*k* = 0.3 W/mK), the largest heating will occur in the filament, i.e., Δ*T* ∼ Δ*T*_f_. Assuming a flat rectangular segment (height *h* = 300 nm; width *w* = 4 μm; Figure 1D), we estimated Δ*T*_f_ = *Ph*/(2*kwL*), where *L* is the segment length and *P* = *GV*^2^ is the dissipative heat generation, with *G* the recorded conductance and *V* the maximum imposed voltage bias. We calculated Δ*T*_f_ at two separate temperatures (300 and 10 K) for the *n* = 16 segments that were investigated at the lowest temperatures. Results are summarized in Table S3.

### Model Parameter Estimation

Transport parameters were determined by applying standard linear and nonlinear least-squares regression in MatLab. The reorganization energy λ was obtained by fitting the relation , where *g*_0_ is a prefactor, via a nonlinear least-squares fit of log(*G*) versus 1/*T*. As a simplification, one can disregard the *T*^–3/2^ dependence, and obtain activation energy *U*_A_ by fitting the relation *G* = *G*_ref_ exp(−*U*_A_/(*k*_B_*T*)), where *G*_ref_ is a prefactor, via a linear least-squares fit of log(*G*) versus 1/*T*. The crossover temperature, *T*_C_ was determined as the first temperature where the conductance *G* as predicted by the Arrhenius fit is 20% lower than the actually measured conductance. The universal scaling curve was fitted via a nonlinear least-squares procedure that adjusts three parameters: the prefactor *B*_0_, the number of sites *N*_S_ and the exponent β.

## Data Availability

All data are publicly available at online digital repository Zenodo^70^ with DOI: 10.5281/zenodo.10656908. The underlying data for each figure are provided with an accompanying metadata description.

## Supporting Information Available

*I*/*V* curves, *G*(*T*,*E*) plots and USC curves for all filaments examined to cryogenic temperatures (Figures S1 and S2), additional data figures (Figure S3–S6), parameters derived from model fitting (Tables S1–S3), and detailed description of model formulations (PDF)

## References

[ref1] Gray H. B.; Winkler J. R. Electron flow through metalloproteins. Biochim. Biophys. Acta, Bioenerg. 2010, 1797, 1563–1572. 10.1016/j.bbabio.2010.05.001. 20460102

[ref2] Moser C. C.; Keske J. M.; Warncke K.; Farid R. S.; Dutton P. L. Nature of biological electron transfer. Nature 1992, 355, 796–802. 10.1038/355796a0. 1311417

[ref3] Page C. C.; Moser C. C.; Chen X.; Dutton P. L. Natural engineering principles of electron tunnelling in biological oxidation–reduction. Nature 1999, 402, 47–52. 10.1038/46972. 10573417

[ref4] Read A. D.; Bentley R. E.; Archer S. L.; Dunham-Snary K. J. Mitochondrial iron–sulfur clusters: Structure, function, and an emerging role in vascular biology. Redox Biol. 2021, 47, 102164 10.1016/j.redox.2021.102164. 34656823 PMC8577454

[ref5] Fleming G.; Martin J.; Breton J. Rates of primary electron transfer in photosynthetic reaction centres and their mechanistic implications. Nature 1988, 333, 190–192. 10.1038/333190a0.

[ref6] Beratan D. N.; Skourtis S. S. Electron transfer mechanisms. Curr. Opin. Chem. Biol. 1998, 2, 235–243. 10.1016/S1367-5931(98)80065-3. 9667934

[ref7] Gray H. B.; Winkler J. R. Long-range electron transfer. Proc. Natl. Acad. Sci. U.S.A. 2005, 102, 3534–3539. 10.1073/pnas.0408029102. 15738403 PMC553296

[ref8] Blumberger J. Recent advances in the theory and molecular simulation of biological electron transfer reactions. Chem. Rev. 2015, 115, 11191–11238. 10.1021/acs.chemrev.5b00298. 26485093

[ref9] Reguera G.; McCarthy K. D.; Mehta T.; Nicoll J. S.; Tuominen M. T.; Lovley D. R. Extracellular electron transfer via microbial nanowires. Nature 2005, 435, 1098–1101. 10.1038/nature03661. 15973408

[ref10] El-Naggar M. Y.; Wanger G.; Leung K. M.; Yuzvinsky T. D.; Southam G.; Yang J.; Lau W. M.; Nealson K. H.; Gorby Y. A. Electrical transport along bacterial nanowires from Shewanella oneidensis MR-1. Proc. Natl. Acad. Sci. U.S.A. 2010, 107, 18127–18131. 10.1073/pnas.1004880107. 20937892 PMC2964190

[ref11] Wang F.; Gu Y.; O’Brien J. P.; Yi S. M.; Yalcin S. E.; Srikanth V.; Shen C.; Vu D.; Ing N. L.; Hochbaum A. I.; et al. Structure of Microbial Nanowires Reveals Stacked Hemes that Transport Electrons over Micrometers. Cell 2019, 177, 361–369.e10. 10.1016/j.cell.2019.03.029. 30951668 PMC6720112

[ref12] Zhang B.; Ryan E.; Wang X.; Song W.; Lindsay S. Electronic transport in molecular wires of precisely controlled length built from modular proteins. ACS Nano 2022, 16, 1671–1680. 10.1021/acsnano.1c10830. 35029115 PMC9279515

[ref13] Huang J.; Zarzycki J.; Gunner M.; Parson W. W.; Kern J. F.; Yano J.; Ducat D. C.; Kramer D. M. Mesoscopic to macroscopic electron transfer by hopping in a crystal network of cytochromes. J. Am. Chem. Soc. 2020, 142, 10459–10467. 10.1021/jacs.0c02729. 32406683

[ref14] Bostick C. D.; Mukhopadhyay S.; Pecht I.; Sheves M.; Cahen D.; Lederman D. Protein bioelectronics: A review of what we do and do not know. Rep. Prog. Phys. 2018, 81, 026601 10.1088/1361-6633/aa85f2. 29303117

[ref15] Bonné R.; Wouters K.; Lustermans J. J.; Manca J. V. Biomaterials and Electroactive Bacteria for Biodegradable Electronics. Front. Microbiol. 2022, 13, 906363 10.3389/fmicb.2022.906363. 35794922 PMC9252516

[ref16] Nielsen L. P.; Risgaard-Petersen N.; Fossing H.; Christensen P. B.; Sayama M. Electric currents couple spatially separated biogeochemical processes in marine sediment. Nature 2010, 463, 1071–1074. 10.1038/nature08790. 20182510

[ref17] Pfeffer C.; Larsen S.; Song J.; Dong M.; Besenbacher F.; Meyer R. L.; Kjeldsen K. U.; Schreiber L.; Gorby Y. A.; El-Naggar M. Y.; et al. Filamentous bacteria transport electrons over centimetre distances. Nature 2012, 491, 218–221. 10.1038/nature11586. 23103872

[ref18] Meysman F. J. Cable bacteria take a new breath using long-distance electricity. Trends Microbiol. 2018, 26, 411–422. 10.1016/j.tim.2017.10.011. 29174100

[ref19] Malkin S. Y.; Rao A. M.; Seitaj D.; Vasquez-Cardenas D.; Zetsche E.-M.; Hidalgo-Martinez S.; Boschker H. T.; Meysman F. J. Natural occurrence of microbial sulphur oxidation by long-range electron transport in the seafloor. ISME J. 2014, 8, 1843–1854. 10.1038/ismej.2014.41. 24671086 PMC4139731

[ref20] Burdorf L. D.; Tramper A.; Seitaj D.; Meire L.; Hidalgo-Martinez S.; Zetsche E.-M.; Boschker H. T.; Meysman F. J. Long-distance electron transport occurs globally in marine sediments. Biogeosciences 2017, 14, 683–701. 10.5194/bg-14-683-2017.

[ref21] Risgaard-Petersen N.; Kristiansen M.; Frederiksen R. B.; Dittmer A. L.; Bjerg J. T.; Trojan D.; Schreiber L.; Damgaard L. R.; Schramm A.; Nielsen L. P. Cable bacteria in freshwater sediments. Appl. Environ. Microbiol. 2015, 81, 6003–6011. 10.1128/AEM.01064-15. 26116678 PMC4551263

[ref22] Geerlings N. M.; Karman C.; Trashin S.; As K. S.; Kienhuis M. V.; Hidalgo-Martinez S.; Vasquez-Cardenas D.; Boschker H. T.; De Wael K.; Middelburg J. J.; Polerecky L.; et al. Division of labor and growth during electrical cooperation in multicellular cable bacteria. Proc. Natl. Acad. Sci. U.S.A. 2020, 117, 5478–5485. 10.1073/pnas.1916244117. 32094191 PMC7071850

[ref23] Bjerg J. T.; Boschker H. T.; Larsen S.; Berry D.; Schmid M.; Millo D.; Tataru P.; Meysman F. J.; Wagner M.; Nielsen L. P.; Schramm A. Long-distance electron transport in individual, living cable bacteria. Proc. Natl. Acad. Sci. U.S.A. 2018, 115, 5786–5791. 10.1073/pnas.1800367115. 29735671 PMC5984516

[ref24] Cornelissen R.; Bøggild A.; Thiruvallur Eachambadi R.; Koning R. I.; Kremer A.; Hidalgo-Martinez S.; Zetsche E.-M.; Damgaard L. R.; Bonné R.; Drijkoningen J.; Geelhoed J. S.; et al. The cell envelope structure of cable bacteria. Front. Microbiol. 2018, 9, 3044 10.3389/fmicb.2018.03044. 30619135 PMC6307468

[ref25] Jiang Z.; Zhang S.; Klausen L. H.; Song J.; Li Q.; Wang Z.; Stokke B. T.; Huang Y.; Besenbacher F.; Nielsen L. P.; Dong M. In vitro single-cell dissection revealing the interior structure of cable bacteria. Proc. Natl. Acad. Sci. U.S.A. 2018, 115, 8517–8522. 10.1073/pnas.1807562115. 30082405 PMC6112711

[ref26] Meysman F. J. R.; Cornelissen R.; Trashin S.; Bonné R.; Martinez S. H.; van der Veen J.; Blom C. J.; Karman C.; Hou J.-L.; Eachambadi R. T.; Geelhoed J. S.; et al. A highly conductive fibre network enables centimetre-scale electron transport in multicellular cable bacteria. Nat. Commun. 2019, 10, 4120–4128. 10.1038/s41467-019-12115-7. 31511526 PMC6739318

[ref27] Thiruvallur Eachambadi R.; Bonné R.; Cornelissen R.; Hidalgo-Martinez S.; Vangronsveld J.; Meysman F. J.; Valcke R.; Cleuren B.; Manca J. V. An ordered and fail-safe electrical network in cable bacteria. Adv. Biosyst. 2020, 4, 2000006 10.1002/adbi.202000006. 32449305

[ref28] Boschker H. T. S.; Cook P. L.; Polerecky L.; Eachambadi R. T.; Lozano H.; Hidalgo-Martinez S.; Khalenkow D.; Spampinato V.; Claes N.; Kundu P.; Wang D.; et al. Efficient long-range conduction in cable bacteria through nickel protein wires. Nat. Commun. 2021, 12, 3996–4012. 10.1038/s41467-021-24312-4. 34183682 PMC8238962

[ref29] Smets B.; Boschker H. T.; Wetherington M. T.; Lelong G.; Hidalgo-Martinez S.; Polerecky L.; Nuyts G.; De Wael K.; Meysman F. J. Multi-wavelength Raman microscopy of nickel-based electron transport in cable bacteria. Front. Microbiol. 2024, 15, 1208033 10.3389/fmicb.2024.1208033. 38525072 PMC10959288

[ref30] Bonné R.; Hou J.-L.; Hustings J.; Wouters K.; Meert M.; Hidalgo-Martinez S.; Cornelissen R.; Morini F.; Thijs S.; Vangronsveld J.; Valcke R.; et al. Intrinsic electrical properties of cable bacteria reveal an Arrhenius temperature dependence. Sci. Rep. 2020, 10, 19798 10.1038/s41598-020-76671-5. 33188289 PMC7666173

[ref31] Schwarze M.; Gaul C.; Scholz R.; Bussolotti F.; Hofacker A.; Schellhammer K. S.; Nell B.; Naab B. D.; Bao Z.; Spoltore D.; Vandewal K.; et al. Molecular parameters responsible for thermally activated transport in doped organic semiconductors. Nat. Mater. 2019, 18, 242–248. 10.1038/s41563-018-0277-0. 30692647

[ref32] De Vault D.; Chance B. Studies of photosynthesis using a pulsed laser: I. Temperature dependence of cytochrome oxidation rate in chromatium. Evidence for tunneling. Biophys. J. 1966, 6, 825–847. 10.1016/S0006-3495(66)86698-5. 5972381 PMC1368046

[ref33] Devault D.; Parkes J. H.; Chance B. Electron tunnelling in cytochromes. Nature 1967, 215, 642–644. 10.1038/215642a0. 6050223

[ref34] Shipps C.; Kelly H. R.; Dahl P. J.; Yi S. M.; Vu D.; Boyer D.; Glynn C.; Sawaya M. R.; Eisenberg D.; Batista V. S.; Malvankar N. S. Intrinsic electronic conductivity of individual atomically resolved amyloid crystals reveals micrometer-long hole hopping via tyrosines. Proc. Natl. Acad. Sci. U.S.A. 2021, 118, e2014139118 10.1073/pnas.2014139118. 33372136 PMC7812754

[ref35] Bera S.; Fereiro J. A.; Saxena S. K.; Chryssikos D.; Majhi K.; Bendikov T.; Sepunaru L.; Ehre D.; Tornow M.; Pecht I.; Vilan A.; et al. Near-Temperature-Independent Electron Transport Well beyond Expected Quantum Tunneling Range via Bacteriorhodopsin Multilayers. J. Am. Chem. Soc. 2023, 145, 24820–24835. 10.1021/jacs.3c09120. 37933117 PMC10655127

[ref36] Geelhoed J. S.; Thorup C. A.; Bjerg J. J.; Schreiber L.; Nielsen L. P.; Schramm A.; Meysman F. J.; Marshall I. P. Indications for a genetic basis for big bacteria and description of the giant cable bacterium Candidatus Electrothrix gigas sp. nov. Microbiol. Spectr. 2023, 11, e0053823 10.1128/spectrum.00538-23. 37732806 PMC10580974

[ref37] Dahl P. J.; Yi S. M.; Gu Y.; Acharya A.; Shipps C.; Neu J.; O’Brien J. P.; Morzan U. N.; Chaudhuri S.; Guberman-Pfeffer M. J.; Vu D.; et al. A 300-fold conductivity increase in microbial cytochrome nanowires due to temperature-induced restructuring of hydrogen bonding networks. Sci. Adv. 2022, 8, eabm7193 10.1126/sciadv.abm7193. 35544567 PMC9094664

[ref38] Gueye M. N.; Carella A.; Faure-Vincent J.; Demadrille R.; Simonato J.-P. Progress in understanding structure and transport properties of PEDOT-based materials: A critical review. Prog. Mater. Sci. 2020, 108, 100616 10.1016/j.pmatsci.2019.100616.

[ref39] Pankratov D.; Hidalgo Martinez S.; Karman C.; Gerzhik A.; Gomila G.; Trashin S.; Boschker H. T.; Geelhoed J. S.; Mayer D.; De Wael K.; JR Meysman F. The organo-metal-like nature of long-range conduction in cable bacteria. Bioelectrochemistry 2024, 157, 108675 10.1016/j.bioelechem.2024.108675. 38422765

[ref40] Egger R.; Mak C.; Weiss U. Quantum rates for nonadiabatic electron transfer. J. Chem. Phys. 1994, 100, 2651–2660. 10.1063/1.466460.

[ref41] Asadi K.; Kronemeijer A. J.; Cramer T.; Jan Anton Koster L.; Blom P. W. M.; de Leeuw D. M. Polaron hopping mediated by nuclear tunnelling in semiconducting polymers at high carrier density. Nat. Commun. 2013, 4, 1710 10.1038/ncomms2708. 23591877

[ref42] Futera Z.; Ide I.; Kayser B.; Garg K.; Jiang X.; Van Wonderen J. H.; Butt J. N.; Ishii H.; Pecht I.; Sheves M.; Cahen D.; et al. Coherent electron transport across a 3 nm bioelectronic junction made of multi-heme proteins. J. Phys. Chem. Lett. 2020, 11, 9766–9774. 10.1021/acs.jpclett.0c02686. 33142062 PMC7681787

[ref43] Eshel Y.; Peskin U.; Amdursky N. Coherence-assisted electron diffusion across the multi-heme protein-based bacterial nanowire. Nanotechnology 2020, 31, 314002 10.1088/1361-6528/ab8767. 32259806

[ref44] van der Veen J. R.; Valianti S.; van der Zant H. S.; Blanter Y. M.; Meysman F. J. A model analysis of centimeter-long electron transport in cable bacteria. Phys. Chem. Chem. Phys. 2024, 26, 3139–3151. 10.1039/D3CP04466A. 38189548

[ref45] Breuer M.; Rosso K. M.; Blumberger J. Electron flow in multiheme bacterial cytochromes is a balancing act between heme electronic interaction and redox potentials. Proc. Natl. Acad. Sci. U.S.A. 2014, 111, 611–616. 10.1073/pnas.1316156111. 24385579 PMC3896160

[ref46] Amdursky N.; Marchak D.; Sepunaru L.; Pecht I.; Sheves M.; Cahen D. Electronic Transport via Proteins. Adv. Mater. 2014, 26, 7142–7161. 10.1002/adma.201402304. 25256438

[ref47] Polizzi N. F.; Skourtis S. S.; Beratan D. N. Physical constraints on charge transport through bacterial nanowires. Faraday Discuss. 2012, 155, 43–61. 10.1039/C1FD00098E. 22470966 PMC3392031

[ref48] Pirbadian S.; Barchinger S. E.; Leung K. M.; Byun H. S.; Jangir Y.; Bouhenni R. A.; Reed S. B.; Romine M. F.; Saffarini D. A.; Shi L.; Gorby Y. A.; et al. Shewanella oneidensis MR-1 nanowires are outer membrane and periplasmic extensions of the extracellular electron transport components. Proc. Natl. Acad. Sci. U.S.A. 2014, 111, 12883–12888. 10.1073/pnas.1410551111. 25143589 PMC4156777

[ref49] Marcus R. A.; Sutin N. Electron transfers in chemistry and biology. Biochim. Biophys. Acta 1985, 811, 265–322. 10.1016/0304-4173(85)90014-X.

[ref50] Hopfield J. Electron transfer between biological molecules by thermally activated tunneling. Proc. Natl. Acad. Sci. U.S.A. 1974, 71, 3640–3644. 10.1073/pnas.71.9.3640. 16592178 PMC433831

[ref51] Jortner J. Temperature dependent activation energy for electron transfer between biological molecules. J. Chem. Phys. 1976, 64, 4860–4867. 10.1063/1.432142.

[ref52] van Grondelle R.; Novoderezhkin V. I. Quantum effects in photosynthesis. Procedia Chem. 2011, 3, 198–210. 10.1016/j.proche.2011.08.027.

[ref53] Richter N.; Chen Z.; Tries A.; Prechtl T.; Narita A.; Müllen K.; Asadi K.; Bonn M.; Kläui M. Charge transport mechanism in networks of armchair graphene nanoribbons. Sci. Rep. 2020, 10, 1988 10.1038/s41598-020-58660-w. 32029795 PMC7005326

[ref54] Kim K. H.; Lara-Avila S.; Kang H.; He H.; Eklof J.; Hong S. J.; Park M.; Moth-Poulsen K.; Matsushita S.; Akagi K.; Kubatkin S.; et al. Apparent power law scaling of variable range hopping conduction in carbonized polymer nanofibers. Sci. Rep. 2016, 6, 37783–37788. 10.1038/srep37783. 27886233 PMC5122886

[ref55] Jortner J.; Ratner M. A. Molecular Electronics; Blackwell Science Oxford, 1997.

[ref56] Filman D. J.; Marino S. F.; Ward J. E.; Yang L.; Mester Z.; Bullitt E.; Lovley D. R.; Strauss M. Cryo-EM reveals the structural basis of long-range electron transport in a cytochrome-based bacterial nanowire. Commun. Biol. 2019, 2, 219–226. 10.1038/s42003-019-0448-9. 31240257 PMC6584659

[ref57] Liu J.; Chakraborty S.; Hosseinzadeh P.; Yu Y.; Tian S.; Petrik I.; Bhagi A.; Lu Y. Metalloproteins containing cytochrome, iron–sulfur, or copper redox centers. Chem. Rev. 2014, 114, 4366–4469. 10.1021/cr400479b. 24758379 PMC4002152

[ref58] Xu S.; Barrozo A.; Tender L. M.; Krylov A. I.; El-Naggar M. Y. Multiheme cytochrome mediated redox conduction through Shewanella oneidensis MR-1 cells. J. Am. Chem. Soc. 2018, 140, 10085–10089. 10.1021/jacs.8b05104. 30056703

[ref59] Malvankar N. S.; Vargas M.; Nevin K. P.; Franks A. E.; Leang C.; Kim B.-C.; Inoue K.; Mester T.; Covalla S. F.; Johnson J. P.; Rotello V. M.; et al. Tunable metallic-like conductivity in microbial nanowire networks. Nat. Nanotechnol. 2011, 6, 573–579. 10.1038/nnano.2011.119. 21822253

[ref60] Amdursky N.; Sepunaru L.; Raichlin S.; Pecht I.; Sheves M.; Cahen D. Electron transfer proteins as electronic conductors: Significance of the metal and its binding site in the blue Cu protein, azurin. Advanced Science 2015, 2, 1400026 10.1002/advs.201400026. 27980928 PMC5115354

[ref61] Matyushov D. V. Reorganization energy of electron transfer. Phys. Chem. Chem. Phys. 2023, 25, 7589–7610. 10.1039/D2CP06072H. 36876860

[ref62] Taylor N. B.; Kassal I. Generalised Marcus theory for multi-molecular delocalised charge transfer. Chem. Sci. 2018, 9, 2942–2951. 10.1039/C8SC00053K. 29732078 PMC5915794

[ref63] Giannini S.; Carof A.; Ellis M.; Yang H.; Ziogos O. G.; Ghosh S.; Blumberger J. Quantum localization and delocalization of charge carriers in organic semiconducting crystals. Nat. Commun. 2019, 10, 3843–3912. 10.1038/s41467-019-11775-9. 31451687 PMC6710274

[ref64] Moser C. C.; Anderson J. R.; Dutton P. L. Guidelines for tunneling in enzymes. Biochim. Biophys. Acta, Bioenerg. 2010, 1797, 1573–1586. 10.1016/j.bbabio.2010.04.441. PMC3509937 20460101

[ref65] Binnig G.; Rohrer H.; Gerber C.; Weibel E. Vacuum tunneling. Phys. B+C 1982, 109–110, 2075–2077. 10.1016/0378-4363(82)90241-8.

[ref66] Frisenda R.; Stefani D.; Van Der Zant H. S. Quantum transport through a single conjugated rigid molecule, a mechanical break junction study. Acc. Chem. Res. 2018, 51, 1359–1367. 10.1021/acs.accounts.7b00493. 29862817

[ref67] Mowat C. G.; Chapman S. K. Multi-heme cytochromes-new structures, new chemistry. Dalton Trans. 2005, 3381–3389. 10.1039/b505184c. 16234915

[ref68] Choi A.; Kim K.; Hong S.; Goh M.; Akagi K.; Kaner R.; Kirova N.; Brazovskii S.; Johnson A.; Bonnell D. A.; Mele E. J.; et al. Probing spin-charge relation by magnetoconductance in one-dimensional polymer nanofibers. Phys. Rev. B: Condens. Matter Mater. Phys. 2012, 86, 155423 10.1103/physrevb.86.155423.

[ref69] Ru X.; Zhang P.; Beratan D. N. Assessing possible mechanisms of micrometer-scale electron transfer in heme-free Geobacter sulfurreducens pili. J. Phys. Chem. B 2019, 123, 5035–5047. 10.1021/acs.jpcb.9b01086. 31095388 PMC6613197

[ref70] van der Veen J. R.; Meysman F. J. R. Characterization of Long-Range Conduction in Cable Bacteria Down to Cryogenic Temperatures [Data Set]; Zenodo, 2024. 10.1021/acsnano.4c12186 PMC11603878 39532345

