# Supplementary material for: Temperature-Dependent Characterization of Long-Range Conduction in Conductive Protein Fibers of Cable Bacteria
Source: ACS Nano. 2024 Nov 12;18(47):32878–89. doi: 10.1021/acsnano.4c12186 (PMC11603878; doi:10.1021/acsnano.4c12186)
Supplement: Supplementary file 1 — nn4c12186_si_001.pdf [file nn4c12186_si_001.pdf]

ACS Nano

Supporting Information

Temperature-dependent characterization of long-range conduction in conductive protein fibers of cable bacteria

Jasper R. van der Veen,[†,‡] Silvia Hidalgo Martinez,[¶] Albert Wieland,[†] Matteo De Pellegrin,[†] Rick Verweij,[†] Yaroslav M. Blanter,[†] Herre S.J. van der Zant,[†] and Filip J.R. Meysman[*,¶,‡]

†*Department of Quantum Nanoscience, Kavli Institute of Nanoscience, Delft University of Technology, Delft 2628 CJ, The Netherlands*

‡*Department of Biotechnology, Delft University of Technology, Delft 2629 HZ, The Netherlands*

¶*Department of Biology, Excellence center for Microbial Systems Technology, University of Antwerp, Wilrijk 2610, Belgium*

E-mail: F.J.R.meysman@tudelft.nl

Supporting figures

Figure S1

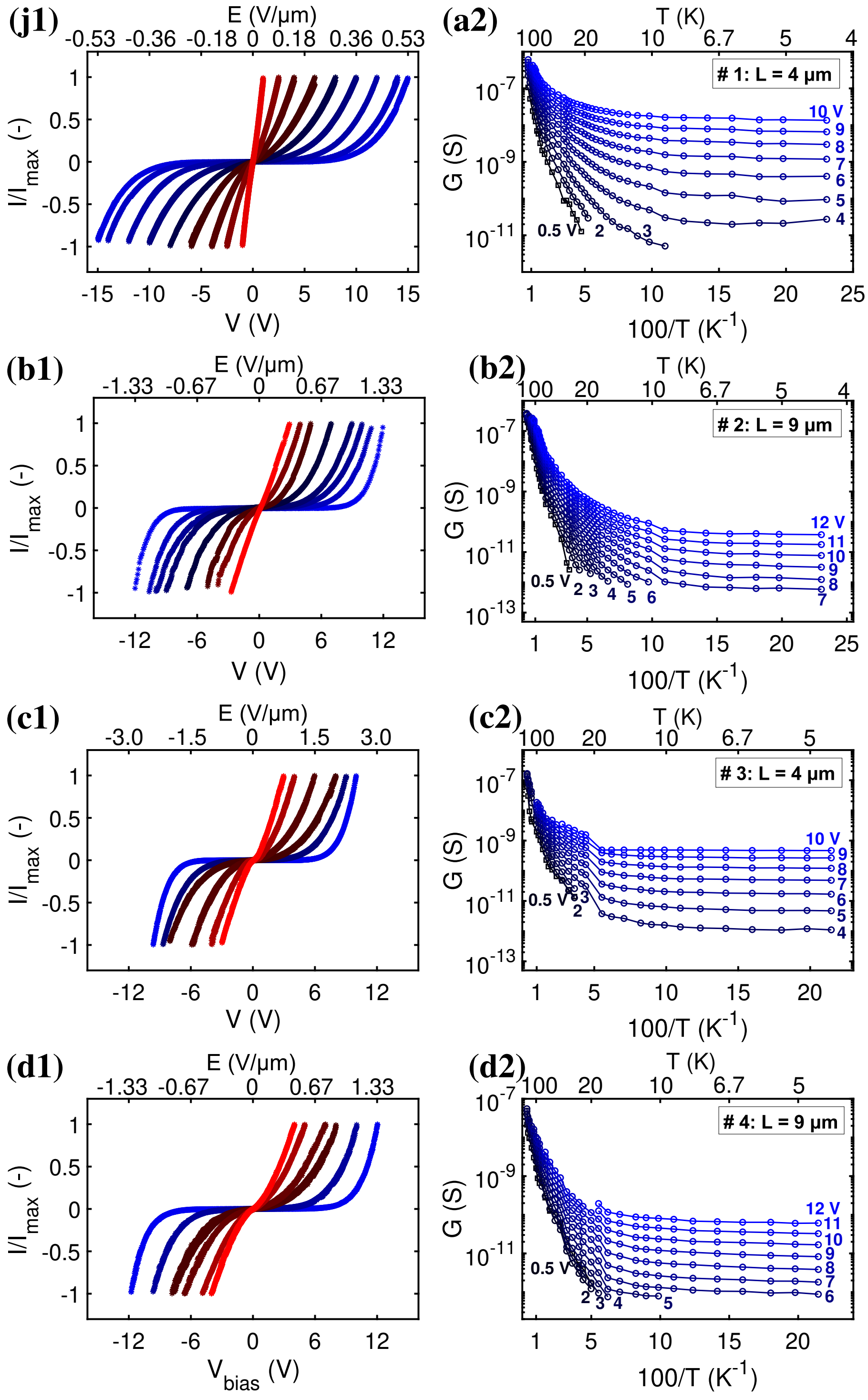

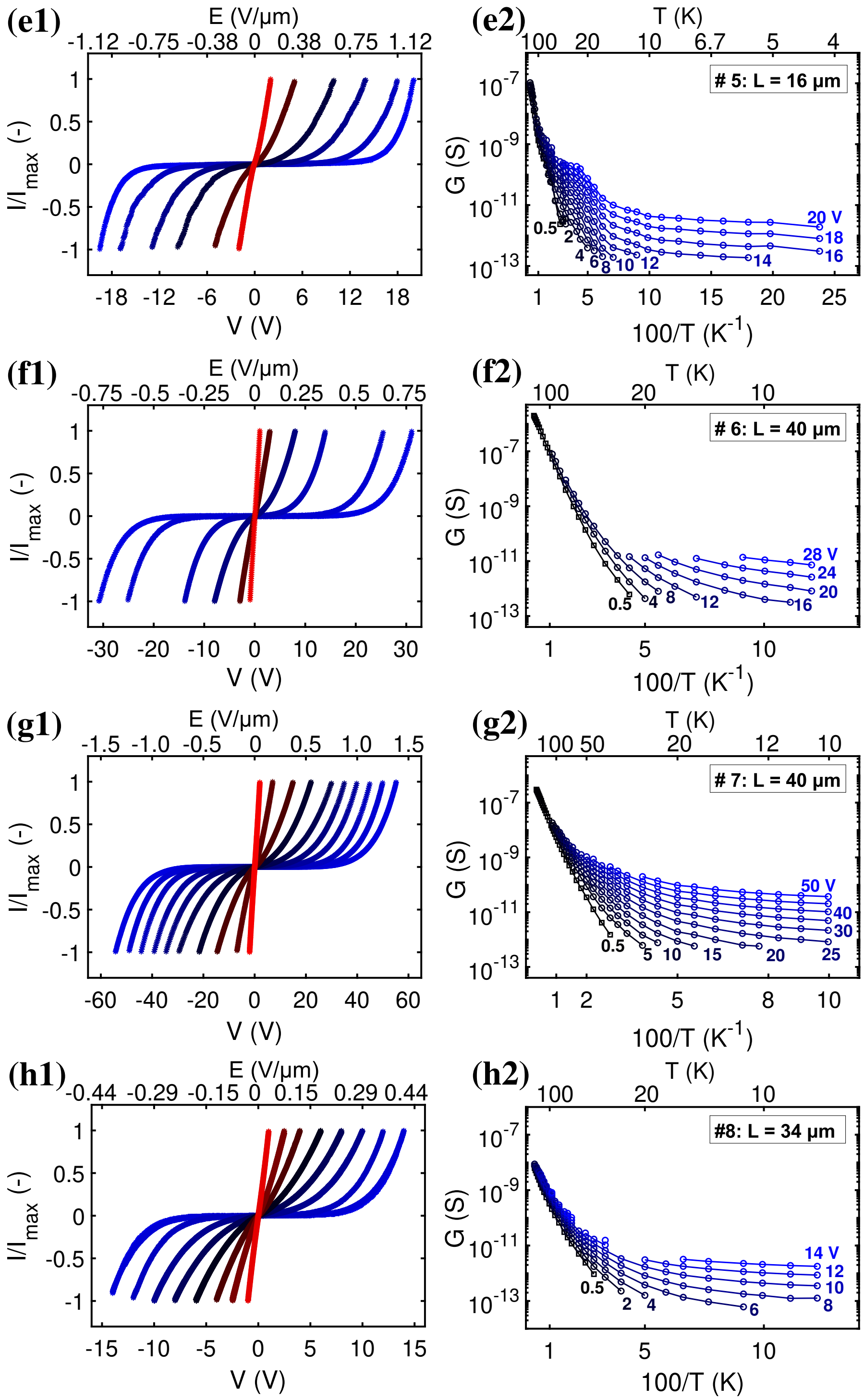
(e1)
E (V/μm)
-1.12 -0.75 -0.38 0 0.38 0.75 1.12
I/I$_{max}$ (-)
V (V)
-18 -12 -6 0 6 12 18
(e2)
T (K)
100 20 10 6.7 5 4
5: L = 16 μm
G (S)
20 V
18
16
14
0.5
2
4
6
8
10
12
100/T (K^{-1})
(f1)
E (V/μm)
-0.75 -0.5 -0.25 0 0.25 0.5 0.75
I/I$_{max}$ (-)
V (V)
-30 -20 -10 0 10 20 30
(f2)
T (K)
100 20 10
6: L = 40 μm
G (S)
28 V
24
20
16
0.5
4
8
12
100/T (K^{-1})
(g1)
E (V/μm)
-1.5 -1.0 -0.5 0 0.5 1.0 1.5
I/I$_{max}$ (-)
V (V)
-60 -40 -20 0 20 40 60
(g2)
T (K)
100 50 20 12 10
7: L = 40 μm
G (S)
50 V
40
30
25
20
15
10
5
0.5
100/T (K^{-1})
(h1)
E (V/μm)
-0.44 -0.29 -0.15 0 0.15 0.29 0.44
I/I$_{max}$ (-)
V (V)
-15 -10 -5 0 5 10 15
(h2)
T (K)
100 20 10
#8: L = 34 μm
G (S)
14 V
12
10
8
6
4
2
0.5
100/T (K)

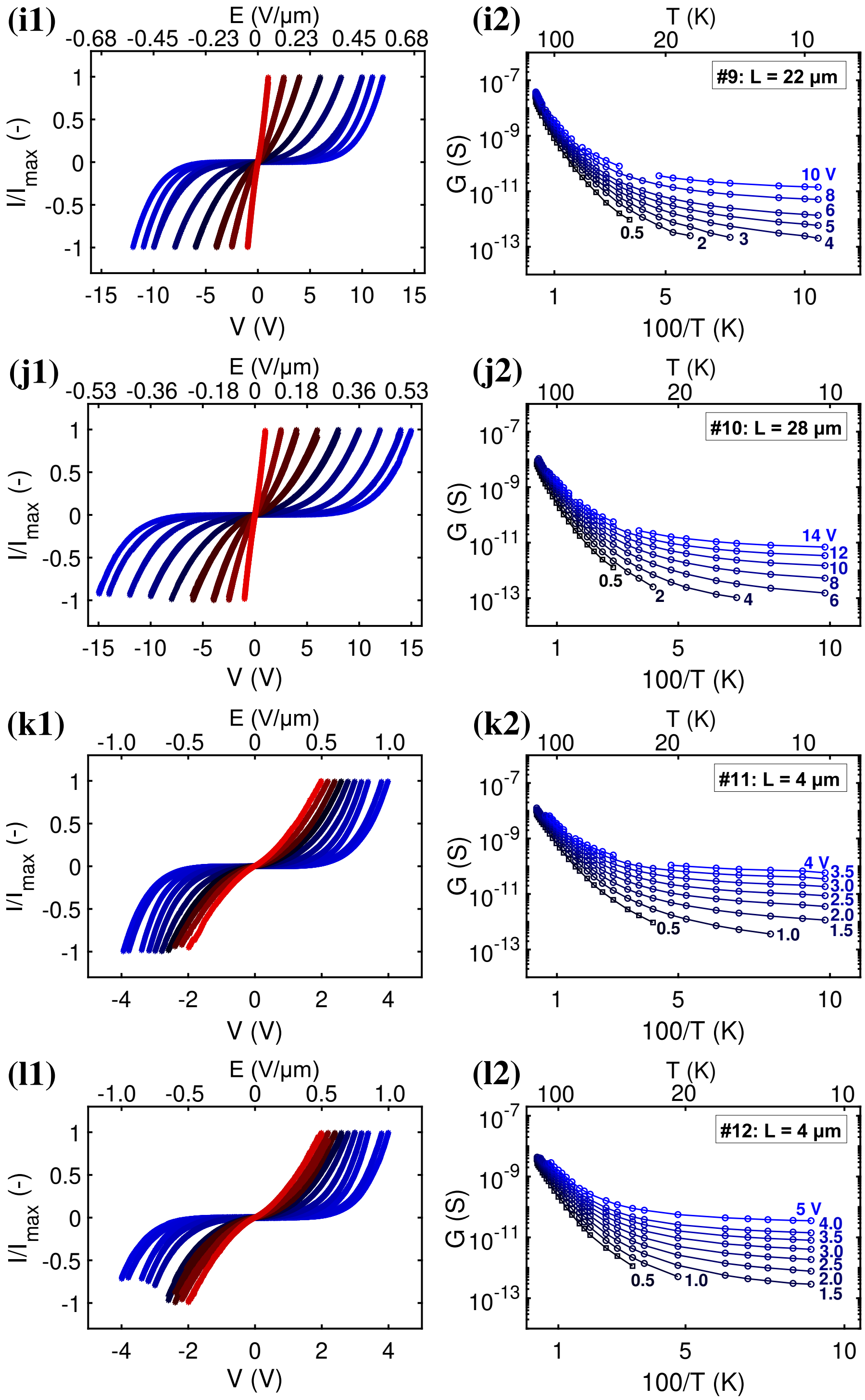
(i1)
E (V/μm)
-0.68 -0.45 -0.23 0 0.23 0.45 0.68
I/I_{max} (-)
V (V)
(i2)
T (K)
100 20 10
#9: L = 22 μm
G (S)
100/T (K)
10 V
8
6
5
4
3
2
0.5
(j1)
E (V/μm)
-0.53 -0.36 -0.18 0 0.18 0.36 0.53
I/I_{max} (-)
V (V)
(j2)
T (K)
#10: L = 28 μm
G (S)
100/T (K)
14 V
12
10
8
6
4
2
0.5
(k1)
E (V/μm)
-1.0 -0.5 0 0.5 1.0
I/I_{max} (-)
V (V)
(k2)
T (K)
#11: L = 4 μm
G (S)
100/T (K)
4 V
3.5
3.0
2.5
2.0
1.5
1.0
0.5
(l1)
E (V/μm)
-1.0 -0.5 0 0.5 1.0
I/I_{max} (-)
V (V)
(l2)
T (K)
#12: L = 4 μm
G (S)
100/T (K)
5 V
4.0
3.5
3.0
2.5
2.0
1.5
1.0
0.5

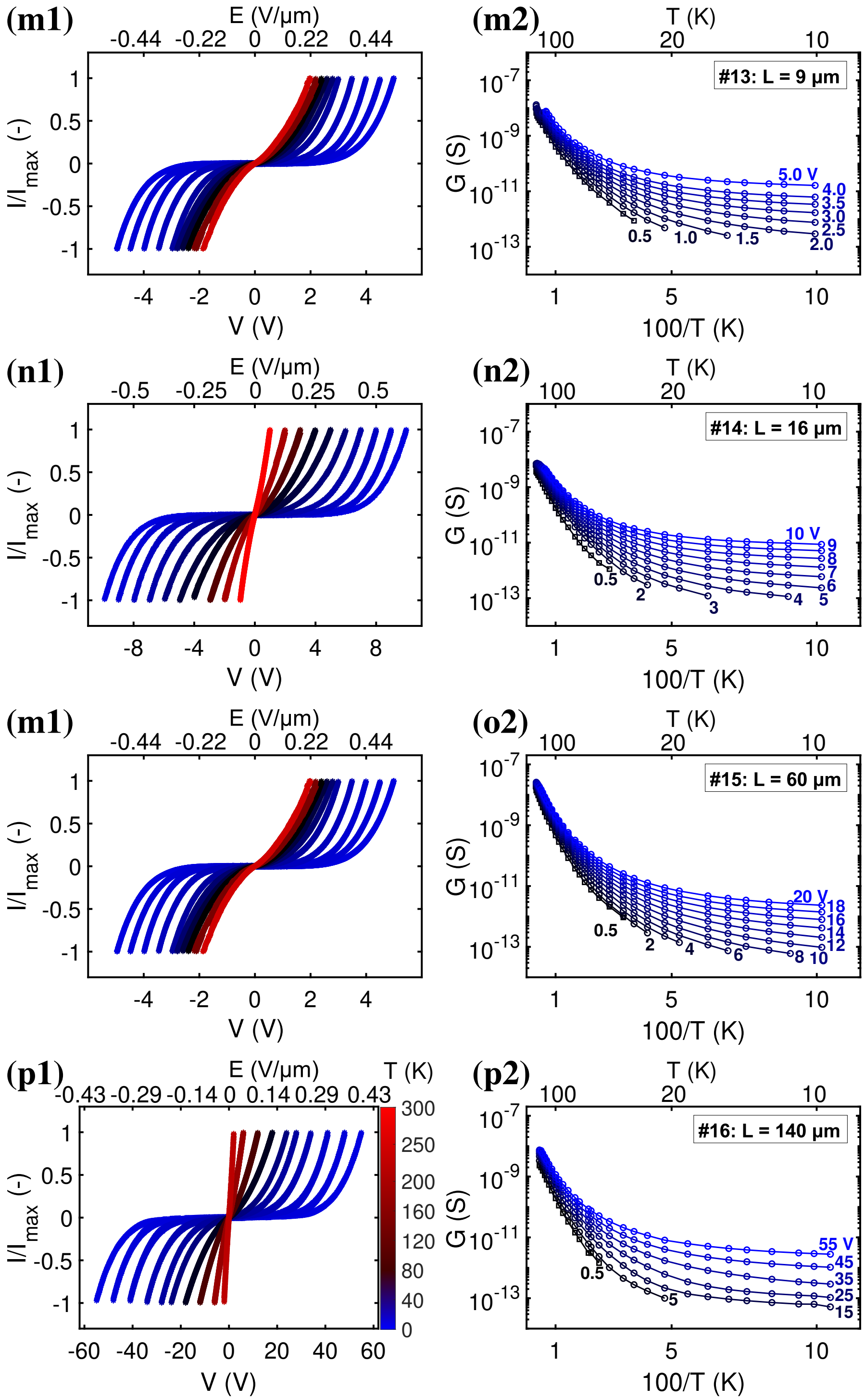
(m1)
E (V/µm)
V (V)
I/I_max (-)
(m2)
T (K)
100/T (K)
G (S)
#13: L = 9 µm
5.0 V
4.0
3.5
3.0
2.5
2.0
0.5
1.0
1.5
(n1)
E (V/µm)
V (V)
I/I_max (-)
(n2)
T (K)
100/T (K)
G (S)
#14: L = 16 µm
10 V
9
8
7
6
5
4
3
2
0.5
(m1)
E (V/µm)
V (V)
I/I_max (-)
(o2)
T (K)
100/T (K)
G (S)
#15: L = 60 µm
20 V
18
16
14
12
10
8
6
4
2
0.5
(p1)
E (V/µm)
T (K)
V (V)
I/I_max (-)
(p2)
T (K)
100/T (K)
G (S)
#16: L = 140 µm
55 V
45
35
25
15
5
0.5

Figure S1. Current/voltage measurements as a function of temperature. The two panels in each row display the data for a given fiber skeleton segment (n= 16 segments of different length L). The segment number and length are indicated in the right panels. (**a1** to **p1**) Two-probe current-voltage ($I(V)$) characteristics recorded at different temperatures. To illustrate the change in shape of the $I(V)$ curve, the current is normalized to the maximum current obtained for each trace. The bottom axis shows the applied voltage bias V, the top axis the applied electric field, $E = V/L$. The colour of the lines indicates the temperature (from red to black in the range $300 - 80$ K and black to blue in the range $80 - 4$ K). See colour bar next to panel (P1). (**a2** to **p2**) The same dataset is replotted in terms of the conductance, $G = I/V$, as a function of the inverse temperature, $100/T$, for different bias voltages, V. The colour scale linearly ranges from black (zero-bias voltage) to blue (maximum bias voltage). Note that the applied voltage range differs per segment (longer segments received higher bias V to attain similar electric field E). The parameters derived for all segments are summarized in Table S1 and S2.

Figure S2

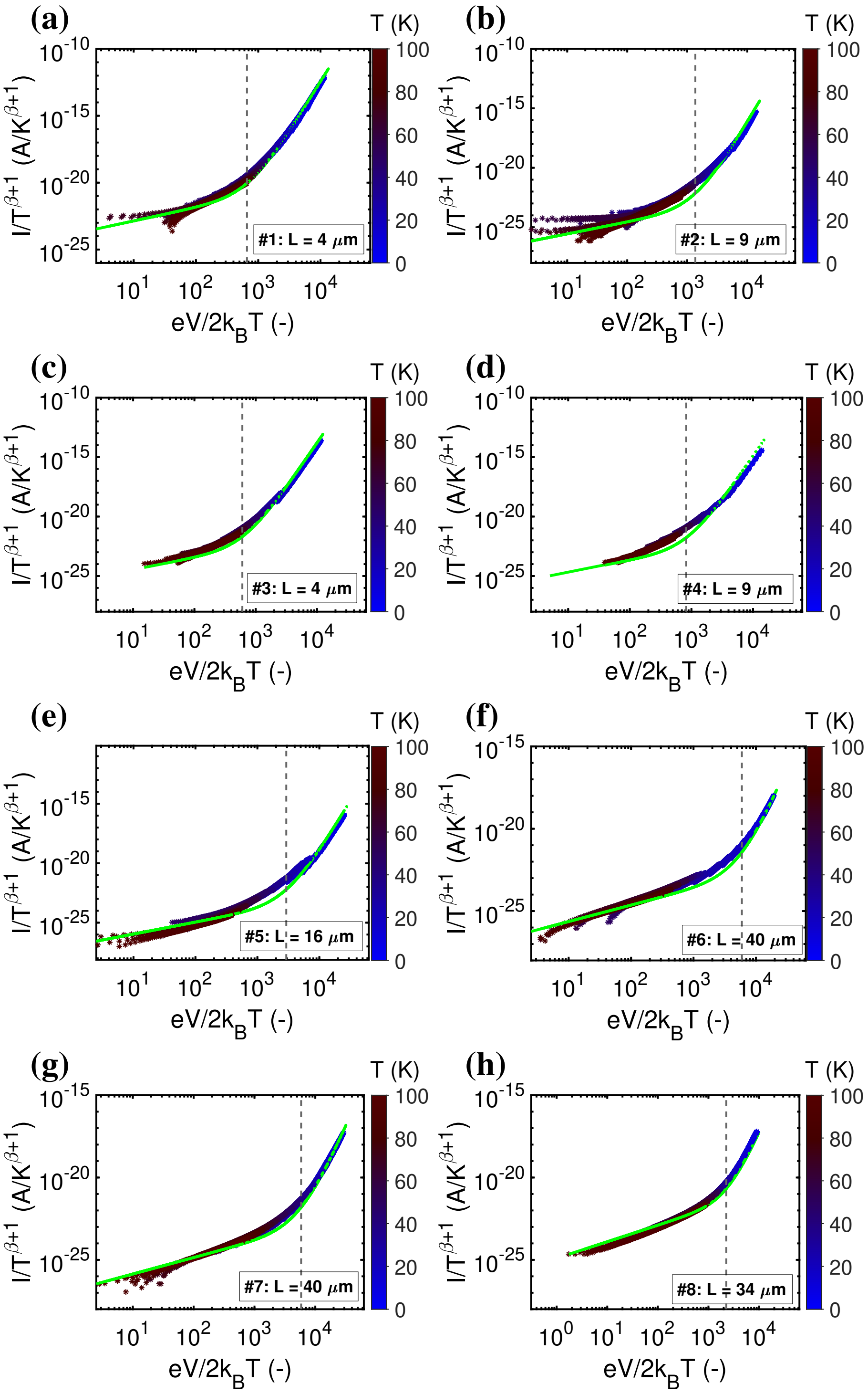

(a)
(b)
(c)
(d)
(e)
(f)
(g)
(h)
#1: L = 4 μm
#2: L = 9 μm
#3: L = 4 μm
#4: L = 9 μm
#5: L = 16 μm
#6: L = 40 μm
#7: L = 40 μm
#8: L = 34 μm
T (K)
$I/T^{\beta+1}$ $(A/K^{\beta+1})$
$eV/2k_BT$ (-)

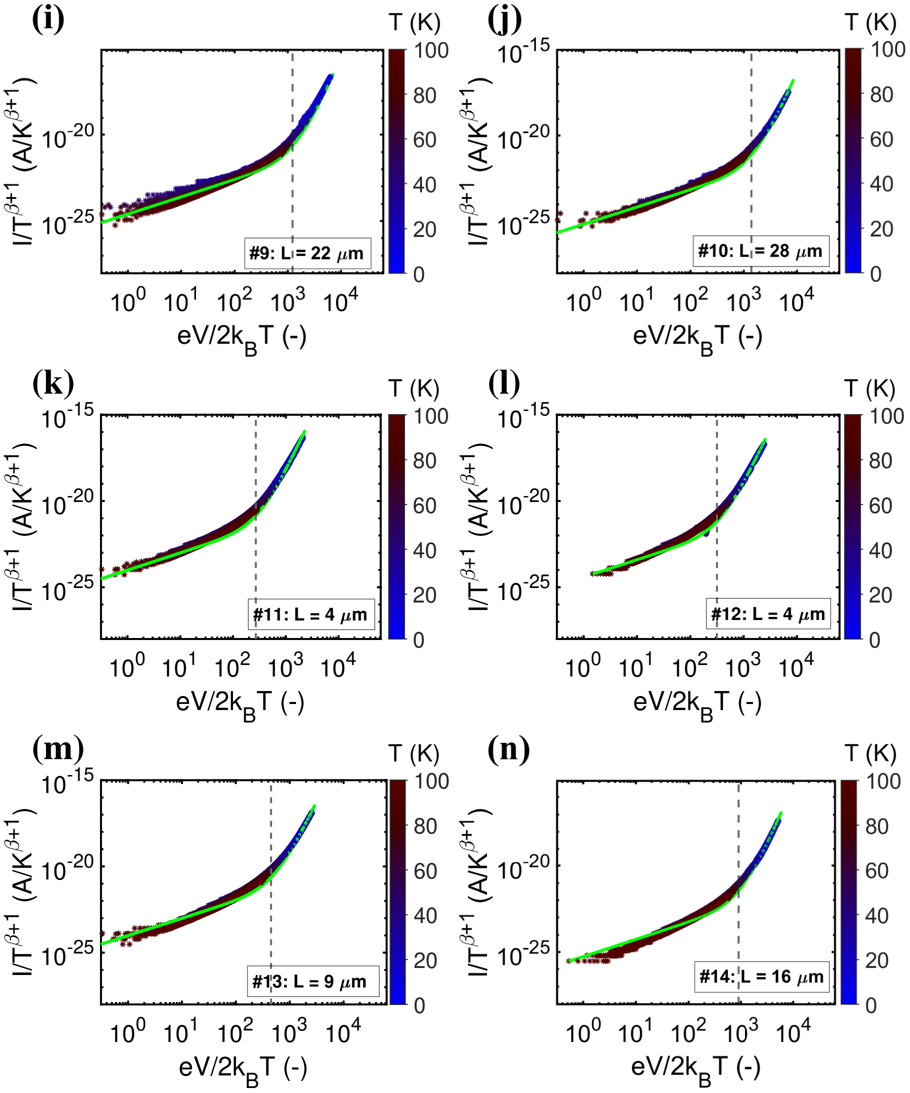

(i)
(j)
(k)
(l)
(m)
(n)
T (K)
#9: L = 22 μm
#10: L = 28 μm
#11: L = 4 μm
#12: L = 4 μm
#13: L = 9 μm
#14: L = 16 μm
I/T^{β+1} (A/K^{β+1})
eV/2k_BT (-)

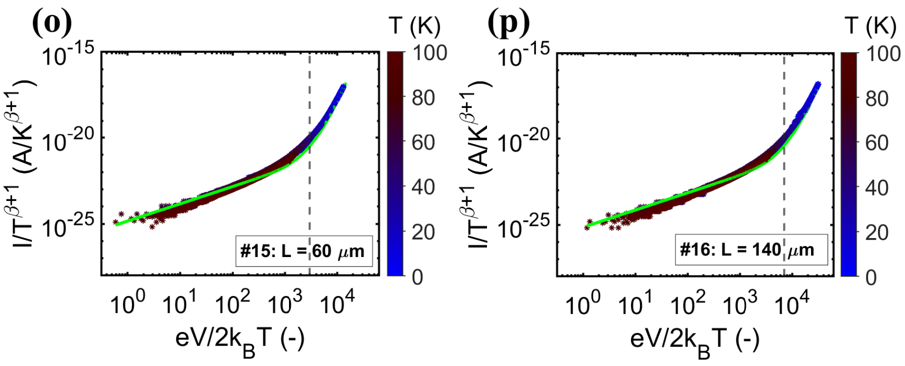

Figure S2. Universal scaling behaviour of the current at low temperatures. (**a** to **p**) Scaling curves are displayed for the 16 segments shown in Figure **S**1. The plots display the scaled current $I/T^{\beta+1}$ on the y-axis versus the scaled bias $eV/2k_{\mathrm{B}}T$ on the x-axis. The $I(V)$ data in the temperature range below 100 K are used. The temperature at which individual data points were collected is indicated by the colour scale next to panel (**O**). The green solid line provides a fit of the universal scaling relation (Eq. 20) to the data. The power law exponent β is visually calibrated to obtain the best fit (precision $\pm$ 0.5). The transition point from the low-bias, high-temperature regime to the high-bias, low-temperature regime occurs at $eV/(2k_{\mathrm{B}}T) \approx N_{\mathrm{S}}$, and is indicated by the dotted line in panel (**O**). The resulting values for the power law exponent, β, and the apparent number of hopping sites, N_{S}, are listed in Table S2.

Figure S3

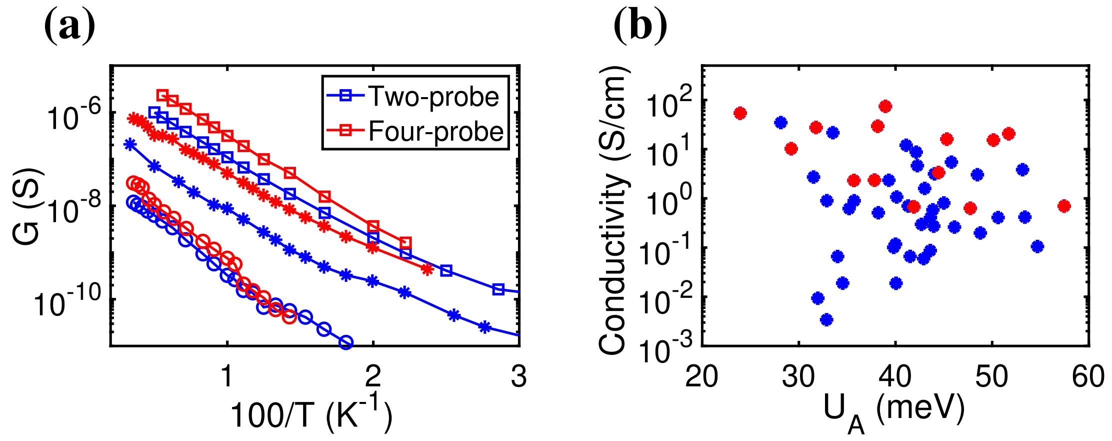

Figure S3. Comparison of four-probe versus two-probe analysis. (**a**) Temperature dependence of the conductance, G, as measured by the two-probe (2P; blue markers) and four-probe (4P; red markers) approach for three different segments. As expected, the 4P conductance is systematically higher as it does not include the contact resistance. The 2P and 4P data show a similar temperature dependence. The activation energy, U_{A}, is determined as the slope from an Arrhenius plot, i.e., a linear fit of ln(G) vs. $1/(k_{\mathrm{B}}T)$ for $T > 100$ K. We find similar activation energies for the 2P and 4P data: segment 1 (squares): 38.1 ± 0.2 meV vs. 38.2 ± 2 meV; segment 2 (stars): 41.2 ± 6.7 meV vs. 35.7 ± 2.3 meV; segment 3 (circles): 48.5 ± 2.3 meV vs. 50.6 ± 3.6 meV. (**b**) Room-temperature fiber conductivity, $\sigma_{0,\mathrm{F}}$ plotted versus activation energy U_{A} for all segments investigated ($n = 53$). Two-probe data (blue markers) and four-probe data (red markers) are provided. The activation energy, U_{A}, and the room temperature conductivity, $\sigma_{0,F}$, are uncorrelated (Pearson correlation coefficient = 0.29). While the variance in $\sigma_{0,F}$ is rather large, the variation in U_{A} is small.

Figure S4

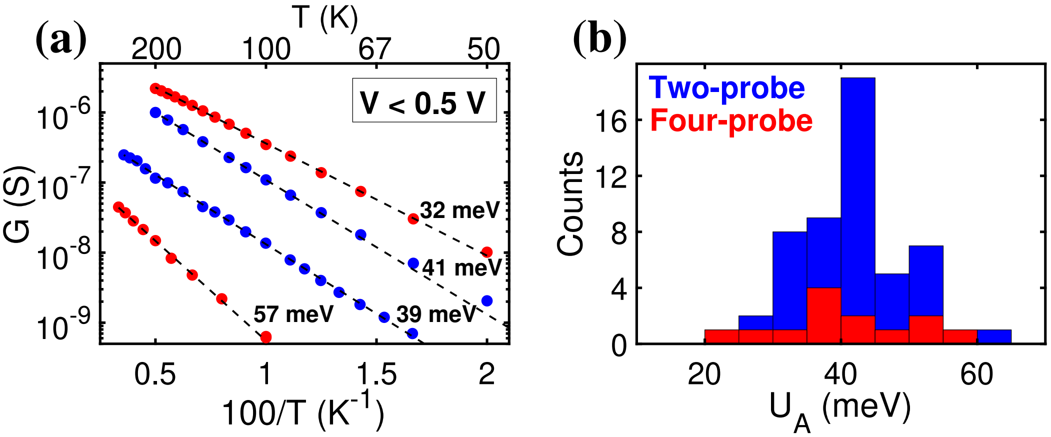

Figure S4. Arrhenius temperature dependence of the conductance. (**a**) Low-bias conductance as a function of temperature plotted above 50 K for two- and four-probe measurements, shown in blue and red respectively. Measurements taken on four different segments. Arrhenius fits (performed for data where $T > 100$ K) and corresponding activation energies are indicated. (**b**) Histogram with the activation energies measured in $n = 53$ segments (blue: two-probe measurements; red: four-probe measurements). The mean activation energy is 42 ± 8 meV ($n = 53$) and the mean goodness of fit across all samples as expressed by the coefficient of determination is $\langle R^2 \rangle \approx 0.982$.

Figure S5

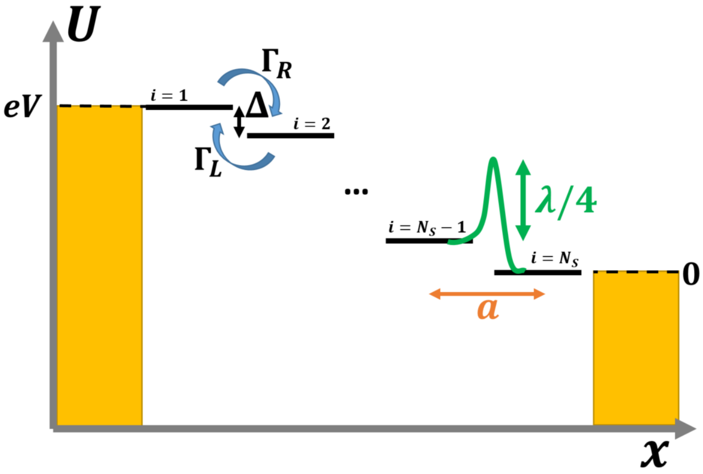

Figure S5. Schematic of a one-dimensional hopping chain. The vertical axis denotes the energy, U, and the horizontal axis the position, x, along the chain. The yellow rectangles represent the electrodes that have an applied voltage bias V ($U = 0$ at the right electrode and $U = eV$ at the left electrode). The horizontal black lines indicate the charge carrier sites, numbered from $i = 1$ to $i = N_\mathrm{S}$, where N_S is the number of sites. The parameter a is the center-to-center distance between adjacent sites. There is an equal energy drop between neighboring sites, $\Delta = eV/N_\mathrm{S}$. There are forward hopping rates, Γ_F, and backwards hopping rates, Γ_B, between any pairs of sites, but only hopping rates between nearest neighbour sites are taken into account. The reorganization energy, λ, determines the activation barrier for hopping (see Supporting Text).

Figure S6

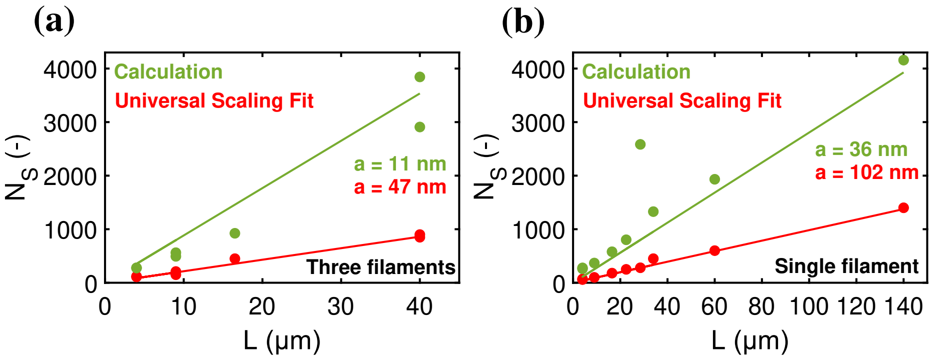

Figure S6. Linear scaling of the number of hopping sites with the segment length. The number of hopping steps, N_S, is determined in two ways. In the first approach (red markers), N_S is derived from the transition point in the universal scaling curves (see Figure SS2). In the second approach (green markers), N_S is calculated via Eq. 31 in the Supporting Text. In both cases, N_S linearly scales with the segment length L (solid lines provide linear fits, excluding offset). The center-to-center distance between hopping sites, a, is given by the slope $a = L/N_\mathrm{S}$. Both methods imply a values that are in excess of 10 nm. (**a**) Data from three independent fiber skeletons. (**b**) Data obtained from different segments on a single fiber skeleton.

Supporting tables

Table S1

Sample	Probes	L (um)	G_0 (nS)	σ_0 (S/cm)	U_A (meV)	G_{ref} (S)	λ (eV)	g_0 (S $eV^{3/2}$)
1	2	4	850	1,1	36,3 ± 3,2	3,12E-06	0,253 ± 0,022	4,06E-08
2	2	40	241	3,0	51,1 ± 1,6	2,37E-06	0,277 ± 0,001	1,61E-08
3	2	240	194	15	59,0 ± 1,6	3,26E-06	0,321 ± 0,015	2,57E-08
4	2	57	15,2	0,27	43,1 ± 1,2	1,77E-07	0,247 ± 0,042	1,16E-09
5	2	70	396	8,7	41,4 ± 1,2	1,73E-06	0,252 ± 0,031	1,50E-08
6	2	16	318	1,6	40,4 ± 2,1	1,26E-06	0,256 ± 0,033	1,24E-08
7	2	9	285	0,81	41,0 ± 2,1	1,31E-06	0,270 ± 0,012	1,52E-08
8	2	4	69,3	0,087	35,1 ± 7,8	1,30E-07	0,258 ± 0,107	1,78E-09
9	2	9	23,3	0,066	31,5 ± 3,8	6,40E-08	0,220 ± 0,027	6,43E-10
10	2	16,5	80,4	0,42	47,1 ± 3,4	4,66E-07	0,298 ± 0,008	5,73E-09
11	2	100	147	4,6	42,3 ± 3,4	6,62E-07	0,316 ± 0,020	1,14E-08
12	2	100	100	3,2	44,0 ± 5,6	4,78E-07	0,323 ± 0,029	8,24E-09
13	2	100	18,7	0,59	43,9 ± 6,8	8,86E-08	0,322 ± 0,044	1,53E-09
14	2	100	3,36	0,11	55 ± 65	2,31E-08	0,365 ± 0,255	3,98E-10
15	2	100	174	5,5	46 ± 12	8,76E-07	0,330 ± 0,062	1,51E-08
16	2	100	28,6	0,90	32,9 ± 3,2	1,01E-07	0,246 ± 0,034	1,24E-09
17	2	100	86,5	2,7	31,5 ± 8,5	2,60E-07	0,273 ± 0,033	4,48E-09
18	2	100	9,45	0,30	42,7 ± 8,1	4,29E-08	0,317 ± 0,050	7,39E-10
19	2	2100	52,7	35	28,1 ± 7,4	1,44E-07	0,259 ± 0,042	2,48E-09
20	4	150	325	15	53,5 ± 1,4	3,14E-06	0,288 ± 0,002	2,22E-08
21	4	50	44,4	0,70	56,7 ± 1,3	3,89E-07	0,327 ± 0,021	3,96E-09
22	4	40	2200	28	31,9 ± 0,4	1,46E-05	0,199 ± 0,002	8,81E-08
23	4	40	4290	54	23,9 ± 1,5	1,01E-05	0,242 ± 0,016	1,74E-07
24	4	80	2950	74	39 ± 15	1,13E-05	0,299 ± 0,077	1,89E-07
25	4	160	46,8	2,4	38 ± 11	1,78E-07	0,298 ± 0,052	3,07E-09
26	4	750	43,5	10	29,2 ± 4,7	1,22E-07	0,263 ± 0,036	2,11E-09
27	4	150	14,3	0,67	42 ± 14	6,25E-08	0,314 ± 0,073	1,08E-09
28	4	255	41,8	3,3	44,5 ± 2,7	2,04E-07	0,324 ± 0,024	3,51E-09
29	4	180	283	16	45,3 ± 9,8	1,41E-06	0,328 ± 0,055	2,44E-08
30	4	180	365	21	52 ± 23	2,23E-06	0,354 ± 0,109	3,86E-08
31	2	50	12,6	0,20	50,7 ± 1,5	1,17E-07	0,277 ± 0,004	8,20E-10
32	2	100	13	0,41	50,5 ± 2,0	7,33E-08	0,287 ± 0,022	6,14E-10
33	4	50	40,1	0,63	54,6 ± 3,1	4,15E-07	0,273 ± 0,021	2,08E-09
34	2	50	39,6	0,62	39,1 ± 1,4	2,32E-07	0,232 ± 0,009	1,65E-09
35	2	30	248	2,3	39,7 ± 0,6	1,30E-06	0,240 ± 0,011	9,97E-09
36	4	10	736	2,3	36,3 ± 1,0	3,34E-06	0,226 ± 0,013	2,55E-08
37	2	10	223	0,70	39,0 ± 2,1	8,10E-07	0,249 ± 0,040	7,84E-09
38	2	9	317	0,90	35,4 ± 1,0	2,48E-06	0,214 ± 0,020	1,55E-08
39	2	40	1720	22	32,2 ± 1,1	5,55E-06	0,219 ± 0,013	4,99E-08
40	2	40	40,7	0,51	37,8 ± 0,4	1,96E-07	0,234 ± 0,010	1,55E-09
41	2	40	32,3	0,41	42,2 ± 1,4	1,76E-07	0,256 ± 0,015	1,47E-09
42	4	40	2330	29	39,3 ± 0,8	3,00E-05	0,225 ± 0,003	1,70E-07
43	2	40	957	12	40,5 ± 0,4	1,01E-05	0,236 ± 0,007	6,42E-08
44	2	40	305	3,8	51,9 ± 0,4	2,38E-06	0,299 ± 0,006	2,15E-08
45	2	34	6,2	0,066	38,5 ± 1,6	2,78E-08	0,249 ± 0,016	2,67E-10
46	2	22,5	16,6	0,12	36,5 ± 1,8	5,86E-08	0,246 ± 0,017	6,15E-10
47	2	28,5	6,67	0,060	39,4 ± 1,7	2,56E-08	0,259 ± 0,016	2,74E-10
48	2	4	7,45	0,0094	30,7 ± 0,7	2,33E-08	0,214 ± 0,011	2,13E-10
49	2	4	2,72	0,0034	31,0 ± 0,9	8,43E-09	0,217 ± 0,012	7,89E-11
50	2	9	6,7	0,019	34,0 ± 1,1	2,22E-08	0,231 ± 0,014	2,12E-10
51	2	16,5	3,64	0,019	38,3 ± 0,6	1,58E-08	0,253 ± 0,006	1,61E-10
52	2	60	13,9	0,26	42,2 ± 1,8	6,30E-08	0,277 ± 0,017	7,30E-10
53	2	140	2,3	0,10	39,5 ± 1,0	1,88E-08	0,240 ± 0,009	1,41E-10

Table 1. Parameters estimated from conductance data within the high temperature range (300 K - 100 K). The first column displays the sample identification number (53 segments have been analysed). L denotes the segment length between two electrode pads. G_0 is the conductance measured at room temperature ($T = 300$ K). σ_0 is the fiber conductivity at $T = 300$ K calculated via Eq.(5) in the main text. The activation energy, U_{A}, and the prefactor, G_{ref}, are determined by fitting the Arrhenius relation (Eq. 11) to the high-temperature conductance data ($T > 100$ K). The reorganization energy, λ, and the prefactor, g_0, are obtained by fitting the Marcus relation (Eq. 10) to the high-temperature conductance data (T > 100 K).

Table S2

Table 2. Parameters estimated from conductance data within the low temperature range (100 K - 4 K). The first column displays the sample identification number (16 segments are analysed). The second column provides the segment ID as listed in Table S1. L is the segment length between two electrode pads and λ is the reorganization energy (both as given in Table S1). The electronic coupling, H, is calculated via Eq. 32. The transition rate at 300 K , Γ_0, is calculated from the Marcus rate expression (Eq. 5) using the values for λ and H listed. The power law exponent, α, was obtained by fitting the temperature-based power law $G \propto T^{\alpha}$ to the conductance data in the region 20 - 100 K (Figure 4c in main text). B_0 is the pre-exponent and β is the power law exponent, obtained by fitting the power law $G = B_0 V^{\beta}$ to the conductance data for $T < 10$ K (Fig. 4d in main text). The characteristic vibration energy, $\hbar\omega_{\mathrm{c}}$, was determined via Eq. 15 from λ and β. The number of sites N_{S} was calculated by Eq. 33 from B_0, λ, β and $\hbar\omega_{\mathrm{c}}$. The corresponding center-to-center distance is $\delta = L/N_{\mathrm{S}}$. Alternatively, the power law exponent β_{USC} and the site number $N_{\mathrm{S,USC}}$ were independently obtained from fitting the universal scaling curves (Figure SS2). The corresponding center-to-center distance between sites is $\delta_{\mathrm{USC}} = L/N_{\mathrm{S,USC}}$.

Sample	ID	L (μm)	λ (meV)	H (meV)	Γ_0 (s^{-1})	α	β	B_0	hω (meV)	N_S	δ (nm)	$\beta_{s,USC}$	$N_{S,USC}$	$\delta_{,USC}$ (nm)
1	1	4	253	5,4	8,3E+10	5,2	7,1	1,10E-15	28	275	15	6,5	120	33
2	7	9	270	4,5	4,8E+10	6,4	8,2	5,23E-20	26	493	18	7,5	210	43
3	8	4	258	1,1	3,5E+09	3,8	6,9	5,51E-17	29	279	14	6,5	110	36
4	9	9	220	0,93	3,7E+09	4,2	6,2	1,08E-17	27	562	16	6,5	150	60
5	10	16,5	298	3,9	2,6E+10	5,1	8,3	2,89E-23	29	924	18	7,5	450	37
6	43	40	236	22	1,6E+12	7,9	6,7	1,56E-21	27	2910	14	8,0	850	47
7	44	40	299	15	4,0E+11	7,0	5,2	6,81E-20	41	3840	10	7,5	900	44
8	45	34	249	0,96	2,8E+09	5,4	4,8	6,95E-18	37	1330	26	6,0	450	76
9	46	22,5	246	1,1	3,9E+09	5,2	4,6	3,10E-16	37	803	28	6,0	250	90
10	47	28,5	259	1,4	5,0E+09	5,1	4,7	3,12E-19	39	2580	11	6,0	280	102
11	48	4	214	0,38	6,4E+08	4,6	4,0	2,27E-13	36	278	14	5,5	60	67
12	49	4	217	0,22	2,2E+08	4,4	4,4	3,34E-14	34	261	15	5,5	70	57
13	50	9	231	0,44	7,3E+08	4,7	4,4	1,45E-14	36	368	24	5,5	100	90
14	51	16,5	253	0,49	7,0E+08	4,9	5,0	9,45E-17	36	579	29	6,0	180	92
15	52	60	277	2,0	8,4E+09	5,2	4,8	1,17E-18	41	1930	31	6,0	600	100
16	53	140	240	1,2	5,0E+09	5,4	5,0	6,58E-21	35	4160	34	6,0	1400	100

Table S3

Table 3. Impact of Joule heating. The first column displays the sample identification number (16 segments as in Table S2). L is the segment length between two electrode pads and V is the maximum applied bias. The measured conductance G, and the calculated dissipative heat generation $P = GV^2$ and associated temperature increase ΔT are given at 300 K and 10 K.

Sample	ID	L (µm)	Max Bias (V)	G (300 K) (nS)	P (300 K) (W)	ΔT (300 K) (K)	G (10 K) (nS)	P (10 K) (W)	ΔT (10 K) (K)
1	1	4	10	850	8,50E-05	2,66E+00	2,00E-08	2,00E-06	6,25E-02
2	7	9	12	285	4,10E-05	5,70E-01	1,00E-10	1,44E-08	2,00E-04
3	8	4	10	69,3	6,93E-06	2,17E-01	6,00E-10	6,00E-08	1,88E-03
4	9	9	12	23,3	3,36E-06	4,66E-02	1,00E-10	1,44E-08	2,00E-04
5	10	16,5	20	80,4	3,22E-05	2,51E-01	2,00E-12	8,00E-10	6,25E-06
6	43	40	28	957	7,50E-04	2,34E+00	1,00E-11	7,84E-09	2,45E-05
7	44	40	50	305	7,63E-04	2,38E+00	8,00E-11	2,00E-07	6,25E-04
8	45	34	14	6,20	1,22E-06	4,47E-03	2,00E-12	3,92E-10	1,44E-06
9	46	22,5	10	16,6	1,66E-06	9,22E-03	3,00E-11	3,00E-09	1,67E-05
10	47	28,5	14	6,67	1,31E-06	5,73E-03	2,00E-11	3,92E-09	1,72E-05
11	48	4	4	7,45	1,19E-07	3,73E-03	1,00E-10	1,60E-09	5,00E-05
12	49	4	5	2,72	6,80E-08	2,13E-03	1,00E-10	2,50E-09	7,81E-05
13	50	9	5	6,70	1,68E-07	2,33E-03	2,00E-11	5,00E-10	6,94E-06
14	51	16,5	10	3,64	3,64E-07	2,76E-03	1,00E-11	1,00E-09	7,58E-06
15	52	60	20	13,9	5,56E-06	1,16E-02	2,00E-12	8,00E-10	1,67E-06
16	53	140	55	2,30	6,96E-06	6,21E-03	1,00E-11	3,03E-08	2,70E-05

Supporting text

1. Models of electron transport in cable bacteria

The models described here form an extension of the hopping model presented in Ref.[1] This reference provides additional details of the basic model assumptions and the model derivation.

1.1. Geometry of the conductive network

Cable bacteria possess a set of N_{F} fiber structures in their cell envelope, which are arranged in parallel and run continuously along the whole length of cm-long filaments[2] (N_{F} = 68; see main text). The fibers themselves consist of a conductive core (diameter d_{F} = 26 nm), surrounded by an electrically insulating shell.[3] The large ratio of length (10^6 nm) to diameter (26 nm) suggests that the electron transport in the fibers is one-dimensional. Still, there could be multiple conduction channels acting in parallel in a single fiber, as d_{F} far exceeds the size of protein monomer as well as the typical spacing between charge carrier sites in metalloproteins ($1 - 2$ nm). At present, it is not known how many conduction channels are actually present. Following Boschker et al.,[3] we assume that a conductive fiber core consists of multiple parallel molecular conduction paths, each $d_{\mathrm{P}} = 4$ nm in diameter. Assuming a hexagonal packing of cylinders, the number of parallel channels in one periplasmic fiber can then be estimated as $N_{\mathrm{C}} = 30$. The total number of parallel paths in a given cable bacterium filament thus amounts to $N_{\mathrm{P}} = N_{\mathrm{F}} \cdot N_{\mathrm{C}} = 2040$. This number of conduction channels should be regarded as a conservative estimate for the electron transfer rate. If less channels are present, then the conductivity of individual channels must be higher, and this in turn also necessitates higher electron transfer rates.

1.2. Current through a single conduction channel

Each conduction channel is modelled as a one-dimensional chain of N_{S} equidistant hopping sites, which serve as temporary localization centers of an electron during the hopping process (Figure SS5). The site at position, i, along the chain is characterised by the site energy, U_{i}. Transitions

between sites are characterised by a forward rate, $\Gamma_{i,j}$, and a backward rate, $\Gamma_{j,i}$. The ratio between forward and backward rates is governed by the detailed balance relation:[4,5]

$$\Gamma_{i,j}/\Gamma_{j,i} = \exp(\Delta_{i,j}/k_B T). \tag{1}$$

Here, k_B is the Boltzmann constant, T is temperature, and $\Delta_{i,j} = U_i - U_j$ is the driving force, i.e., the difference between the site energy of the initial position of the electron, U_i, and that of its final position, U_j. In our model, only nearest neighbour hopping is considered ($j = i \pm 1$). Moreover, the hopping sites are assumed to be identical and periodic in structure, with the same center-to-center distance a between them (Figure SS5). As sites are identical, the transition rate is the same for every pair of sites (forward $\Gamma_{i,i+1} = \Gamma_F$; backward $\Gamma_{i+1,i} = \Gamma_B$). Moreover, if we connect the hopping chain to an electrode on either side, and impose a voltage bias V between the electrodes, the energy difference between two sites is constant and amounts to $\Delta_{i,j} = \Delta = eV/N_S$ (assuming there is no voltage drop over the electrode interface). The current between two consecutive hopping sites along a single conduction path is given by:

$$I_P = e\left[\Gamma_F p_i \left(1 - p_{i+1}\right) - \Gamma_B p_{i+1}\left(1 - p_i\right)\right], \tag{2}$$

where p_i and p_{i+1} are the occupation probabilities at the consecutive hopping sites, and e is the elementary charge (i.e., the magnitude of the charge carried by a single electron). This expression embeds the Pauli exclusion principle: an electron can only jump to a charge carrier site when this site is vacant.

When fiber skeletons are connected to electrodes and electrically investigated at room temperature, the resulting I/V curves are conspicuously linear. A comparison of model-simulated and experimentally recorded I/V curves indicates that the charge transport is field-driven regime rather than concentration-driven, with no significant injection barrier (see detailed discussion in Ref.[1]). In this field-driven scenario, the site occupancy does not show a gradient ($p_i = 1/2$) and so electron transport is solely "drift" based, i.e., it is driven by the difference in the forward and backward

transition rates:

$$I_{\rm P} = \frac{e}{4}\left[\Gamma_{\rm F} - \Gamma_{\rm B}\right], \tag{3}$$

Accounting for $N_{\rm P}$ parallel conduction channels, and implementing the detailed balance relation, Eq. 1, the total current along a single filament, can be expressed as:

$$I = N_{\rm P} I_{\rm P} = \frac{e}{4} N_{\rm P}\Gamma_{\rm F}\left(1 - \exp(-\Delta/k_{\rm B}T)\right). \tag{4}$$

By implementing suitable expressions for the transition rate, $\Gamma_{\rm F}$, a set of separate hopping models can be developed.

2. Transition rate models

At low temperatures, the energy stored in vibrational modes can stimulate the hopping process. To account for this effect, several authors have formulated an extended, quantum version of the semi-classical Marcus theory of electron transfer. These models explicitly account for vibrational modes that suitably couple to the electron transfer. Two prominent examples are the single effective mode formulation presented by Jortner[6] and the multiple mode formulation developed by Egger et al.[4] Below, we will integrate these two vibrational model formulations in the multistep hopping chain model introduced above. But first, we introduce the classical Marcus rate formalism as a reference for these more elaborate quantum-based models.

2.1. Standard non-adabiatic electron transfer: the Marcus formulation

At sufficiently high temperatures, the thermal energy, $k_{\rm B}T$, exceeds the quantum energy, $\hbar\omega$, of the intramolecular vibrations, and all vibrations are thermally excited. Under these conditions, the transition rate between neighbouring sites is given by the classical Marcus rate expression,[7]

$$\Gamma_{\rm F} = \frac{2\pi}{\hbar}\frac{H^2}{\sqrt{4\pi\lambda k_{\rm B}T}}\exp\left[\frac{-(\lambda-\Delta)^2}{4\lambda k_{\rm B}T}\right]. \tag{5}$$

Here, λ is the reorganization energy, H is the electronic coupling, and $\Delta = eV/N_{\rm S}$ is the energy difference between adjacent sites as introduced above. Because the bacterial filaments investigated are very long, the number of hopping sites N_S is large, and so the driving force Δ is smaller than the reorganization energy, $\lambda \approx 270$ meV, for the bias voltages V applied. To illustrate this: if we impose a 1 V bias across the smallest segment investigated ($L = 4\ \mu$m) and assume a hopping distance of $a \sim 0.7$ nm similar to that of OmcS nanowires,[8] we obtain $\Delta = e\delta V/L = 0.17$ meV. Under these conditions ($\Delta \ll \lambda$), the approximation $(\lambda - \Delta)^2 \approx \lambda(\lambda - 2\Delta)$ holds, and the transition rate becomes

$$\Gamma_{\rm F} = \frac{2\pi}{\hbar}\frac{H^2}{\sqrt{4\pi\lambda k_{\rm B}T}}\exp\left[-\frac{\lambda}{4k_{\rm B}T}\right]\exp\left[\frac{\Delta}{2k_{\rm B}T}\right]. \tag{6}$$

Upon substitution of this expression into Eq. 4, we can write the current:

$$I = N_{\rm P}\frac{e\pi}{\hbar}\frac{H^2}{\sqrt{4\pi\lambda k_{\rm B}T}}\exp\left[-\frac{\lambda}{4k_{\rm B}T}\right]\sinh\left(\frac{\Delta}{2k_{\rm B}T}\right). \tag{7}$$

Accordingly, at a given temperature, the I/V curve is predicted to attain a sinh-type shape. Using $\Delta = e\delta E$, the corresponding conductance $G = I/V$ can be expressed as:

$$G(T,E) = N_P\frac{e\pi}{\hbar L}\frac{H^2}{\sqrt{4\pi\lambda k_{\rm B}T}}\exp\left(-\frac{\lambda}{4k_{\rm B}T}\right)\frac{1}{E}\sinh\left(\frac{e\delta E}{2k_{\rm B}T}\right). \tag{8}$$

Because the bacterial filaments investigated are long and so N_S is large, the driving force Δ is also smaller than the thermal energy scale $k_{\rm B}T$ for high enough temperatures. Under these conditions, when $\Delta = e\delta E \ll k_{\rm B}T$, the above expression becomes independent of the electric field, and simplifies to:

$$G(T) = g_0(k_{\rm B}T)^{-3/2}\exp\left(-\frac{\lambda}{4k_{\rm B}T}\right). \tag{9}$$

This equation was fitted to the $G(T)$ data within the high-temperature regime to provide values for the reorganization energy λ (tabulated in Table S1). The prefactor g_0 is given by:

$$g_0 = \frac{N_P}{N_S}\frac{e^2\sqrt{\pi}}{4\hbar}\frac{H^2}{\sqrt{\lambda}}. \tag{10}$$

However, it is known that the electronic coupling H is weakly dependent on temperature.[9] As a simplification, one can disregard the $T^{-3/2}$ dependence to obtain the classical Arrhenius expression:

$$G(T) = G_{\text{ref}}\exp\left(-\frac{U_{\text{A}}}{k_{\text{B}}T}\right). \tag{11}$$

The prefactor $G_{\text{ref}} = g_0(k_{\text{B}}T)^{-3/2}$ is now considered to be a temperature-independent parameter. This equation was fitted to the $G(T)$ data within the high-temperature regime to provide values for the activation energy U_A (tabulated in Table S1). The activation and reorganization energy are related through $U_{\text{A}} = \lambda/4$, which is a classical result from Marcus theory.[10]

2.2. One effective vibrational mode: the Jortner model formulation

When the quantum energy of vibrations $\hbar\omega$ becomes smaller than the thermal energy, $k_{\text{B}}T$, the Marcus rate expression (Eq. 5) is no longer valid. In the Jortner model formulation, vibrational modes are characterised by a single effective mode $\langle\omega\rangle$, which represents a suitable average across all molecular vibrations. The corresponding forward transition rate becomes:[6]

$$\Gamma_{\text{F}} = \Gamma_0 \cdot \exp(-S(2\overline{n}_{\text{B}}+1)) \cdot I_{\text{q}}(2S(\overline{n}_{\text{B}}(\overline{n}_{\text{B}}+1))^{1/2}) \cdot (\overline{n}_{\text{B}}(\overline{n}_{\text{B}}+1))^{q/2}. \tag{12}$$

In this expression, Γ_0 is a temperature-independent pre-factor, $S = \lambda/\hbar\langle\omega\rangle$ is the electron-phonon coupling strength associated with $\langle\omega\rangle$, I_{q} is the modified Bessel function, $\overline{n}_{\text{B}} = 1/(\exp(\hbar\langle\omega\rangle/k_{\text{B}}T) - 1)$ is the Bose-Einstein distribution for the single effective mode $\langle\omega\rangle$, and the exponent $q = \Delta/\hbar\langle\omega\rangle$ denotes the ratio between the site energy drop between hopping sites and the mean quantum vibrational energy.[6] One can show that at high temperatures (when $\hbar\langle\omega\rangle \ll k_{\text{B}}T$), the Marcus rate

expression Eq. 5 is recovered .[6] The Jortner expression for the transition rate, Eq. 12, can be combined with the expression for the current along a single filament, Eq. 4, in order to derive the conductance $G(T, E)$ as a function of temperature and the electric field strength.

2.3. Multiple vibrational modes: the Egger model formulation

In the Egger model formulation,[4] it is assumed that many vibrational modes are coupled to the electron hopping process. The phonon spectral density function $J(\omega)$ describes how much a certain vibrational mode contributes to the reorganization energy:[11]

$$\lambda = \hbar \int_0^\infty \omega J(\omega) \mathrm{d}\omega. \tag{13}$$

The Egger model formulation assumes that the phonon spectral density function has an Ohmic form:

$$J(\omega) = (\beta + 2) \frac{e^{-\omega/\omega_\mathrm{c}}}{\omega}. \tag{14}$$

Here, ω_c is the characteristic frequency of the spectrum. The parameter β provides a measure for the electron-phonon coupling strength. For the Ohmic spectral density, the reorganization energy is directly proportional to the characteristic frequency, ω_c, as stated in the original derivation of the Egger model:[4]

$$\lambda = (\beta + 2)\hbar\omega_\mathrm{c}, \tag{15}$$

Since the spectrum starts at zero frequency, some modes remain thermally activated at the lowest temperatures. This explains an important difference between the Jortner and Egger model formulations. Whereas in the Jortner model, the low-bias conductance remains constant at cryogenic temperatures, this is not the case in the Egger model: the low-bias conductance shows a power law behaviour, in which the conductance further decreases as the temperature decreases. Effectively, the modes that remain thermally activated at low temperatures force the low-bias conductance to die out.

When the temperature is much lower than the characteristic energy of vibration ($k_\mathrm{B}T \ll \hbar\omega_\mathrm{c}$),

the Egger model provides following expression for the transition rate - see Eq.3.6 in:[4]

$$\Gamma_{\mathrm{F}} = \frac{1}{\hbar}\frac{H^2}{\gamma(\beta+2)\hbar\omega_{\mathrm{c}}}\left(\frac{2\pi k_{\mathrm{B}}T}{\hbar\omega_{\mathrm{c}}}\right)^{\beta+1}\left|\gamma\left(1+\beta/2+i\frac{eV}{2\pi N_{\mathrm{S}}k_{\mathrm{B}}T}\right)\right|^2 \exp\left(\frac{eV}{2N_{\mathrm{S}}k_{\mathrm{B}}T}\right)\exp\left(-\frac{eV}{N_{\mathrm{S}}\hbar\omega_{\mathrm{c}}}\right). \tag{16}$$

Here, γ is the complex gamma function. If we combine the expression for the current along a single filament, Eq. 4, with the above expression for the transition rate, Eq. 16, we obtain:

$$I = A_0 T^{\beta+1}\sinh\left(\frac{eV}{2N_{\mathrm{S}}k_{\mathrm{B}}T}\right)\exp\left(-\frac{eV}{N_{\mathrm{S}}\hbar\omega_{\mathrm{c}}}\right)\left|\gamma\left(1+\beta/2+i\frac{eV}{2\pi N_{\mathrm{S}}k_{\mathrm{B}}T}\right)\right|^2. \tag{17}$$

The pre-factor A_0 combines all temperature-independent parameters:

$$A_0 = N_P\frac{e}{2\hbar}\frac{H^2}{\gamma(\beta+2)\hbar\omega_{\mathrm{c}}}\left(\frac{2\pi k_{\mathrm{B}}}{\hbar\omega_{\mathrm{c}}}\right)^{\beta+1}. \tag{18}$$

Note, that the Egger model centrally depends on the assumption that the phonon spectral density function has an Ohmic form. At present, the details of electron-phonon coupling in the conductive fibers of cable bacteria are unknown, and so, alternative shapes phonon spectral density function could be relevant.[11]

2.4. Egger model: Universal scaling of current

We can assume that the number of hopping events N_S needed for charge carriers to traverse the distance between the electrodes is high, so that $eV/N_S \ll \hbar\omega_{\mathrm{c}}$. As detailed in the main text, eV/N_S is on the order of 0.2 meV or lower, for the segments investigated, while $\hbar\omega_{\mathrm{c}}$ ranges between 26-41 meV. Hence, this condition appears to be satisfied, and as a result, the exponential term containing $eV/(N_S\hbar\omega_{\mathrm{c}})$ vanishes in the current relation of the Egger model.

$$I = A_0 T^{\beta+1}\sinh\left(\frac{eV}{2N_{\mathrm{S}}k_{\mathrm{B}}T}\right)\left|\gamma\left(1+\beta/2+i\frac{eV}{2\pi N_{\mathrm{S}}k_{\mathrm{B}}T}\right)\right|^2. \tag{19}$$

This $I(V)$ curve can be normalized by scaling the bias voltage as $\overline{V} = eV/(2k_\mathrm{B}T)$ (the horizontal axis) and rescaling the current as $I/T^{\beta+1}$ (the vertical axis).

$$\overline{I} = A_0 \sinh\left(\frac{\overline{V}}{N_\mathrm{S}}\right)\left|\gamma\left(1+\beta/2+i\frac{\overline{V}}{\pi N_\mathrm{S}}\right)\right|^2. \tag{20}$$

If the Egger model applies, then the $I(V)$ data collected at different temperatures should fall onto a single curve, only depending on the prefactor A_0, the exponent β, and the number of hopping sites N_S. These universal scaling plots are shown for all segments investigated at low temperature in Figure S**S**2. At low values of $\overline{V}$, the scaled current increases linearly with the normalized bias. At high values of $\overline{V}$, the scaled current increases superlinearly. There is a single crossover point that depends on the number of hopping sites N_S.

2.5. Egger model: universal scaling of conductance

Starting from the simplified current expression of the Egger model, Eq. 20, the associated dependency of the conductance on temperature and electric field becomes:

$$G(T,E) = \left(\frac{eA_0}{2N_S k_\mathrm{B}}\right)T^\beta\left(\frac{2k_\mathrm{B}T}{e\delta E}\right)\sinh\left(\frac{e\delta E}{2k_\mathrm{B}T}\right)\left|\gamma\left(1+\beta/2+i\frac{e\delta E}{2\pi k_\mathrm{B}T}\right)\right|^2. \tag{21}$$

This $G(T,E)$ relation can be normalized by scaling the electric field as $\overline{E} = eLE/(2k_\mathrm{B}T)$ (the horizontal axis) and rescaling the conductance as $\overline{G} = G/T^\beta$ (the vertical axis).

$$\overline{G}(T,\overline{E}) = \left(\frac{eA_0}{2N_S k_\mathrm{B}}\right)\frac{\sinh(\overline{E}/N_S)}{(\overline{E}/N_S)}\left|\gamma\left(1+\beta/2+i\frac{\overline{E}}{\pi N_S}\right)\right|^2. \tag{22}$$

If $\beta/2$ is an integer, then the real part of the gamma function in Eq. 22 can be expressed in the closed form:

$$\left|\gamma\left(1+\beta/2+i\frac{x}{\pi}\right)\right|^2 = \frac{x}{\sinh(x)}\prod_{k=1}^{\beta/2}\left(k^2+\left(\frac{x}{\pi}\right)^2\right) \tag{23}$$

As a result, the conductance can be rewritten as:

$$\overline{G}(T,\overline{E}) = \left(\frac{eA_0}{2N_S k_\mathrm{B}}\right) \prod_{k=1}^{\beta/2} \left(k^2 + \left(\frac{\overline{E}}{\pi N_S}\right)^2\right). \tag{24}$$

The rescaled conductance shows two asymptotic regimes. In the low-bias, high-temperature regime ($eV/(2N_\mathrm{S}) < k_\mathrm{B}T \ll \hbar\omega_\mathrm{c}$), we can take the limit for $\overline{E} \to 0$, and obtain:

$$\overline{G}(T,\overline{E}) = \left(\frac{eA_0}{2N_S k_\mathrm{B}}\right) \left|\gamma\left(1+\beta/2\right)\right|^2. \tag{25}$$

Accordingly, the rescaled conductance becomes independent of the electric field $\overline{E}$. This implies that the conductance should follow a power law dependence on temperature, $G \propto T^\beta$. This is seen in the data (Fig. 4a in main text). Likewise, in the high-bias, low-temperature regime ($k_\mathrm{B}T < eV/(2N_\mathrm{S}) \ll \hbar\omega_\mathrm{c}$), the dominant term for large $\overline{E}$ becomes:

$$\overline{G}(T,\overline{E}) = \left(\frac{eA_0}{2N_S k_\mathrm{B}}\right) \left(\frac{\overline{E}}{\pi N_S}\right)^\beta. \tag{26}$$

Accordingly, the rescaled conductance shows a power law dependence on the rescaled electric field, $G \propto E^\beta$. This strong dependence on the electric field is indeed seen in the data (Fig. 4b in main text) for low temperatures $T < 10$ K.

The inflection point between the two regimes is dependent on the number of sites N_S in the hopping chain, and is determined by point where $\overline{G}(T,\overline{E})$ value in Eq. 25 attains the same value as Eq. 26:

$$\left(\frac{\overline{E}}{\pi N_S}\right)^\beta = \left|\gamma\left(1+\beta/2\right)\right|^2. \tag{27}$$

One can easily show that this condition is satisfied when

$$\overline{E} = (\beta - 1)N_S. \tag{28}$$

3. The distance between hopping sites

Each conduction channel is modelled as a one-dimensional chain of N_S equidistant hopping sites with the same center-to-center distance $\delta = L/N_\mathrm{S}$ between them (Figure SS5). We can estimate the number of hopping sites N_S from our data in two independent ways. In a first approach, we can extract N_S directly from the universal scaling curve. The scaling curves show a pronounced inflection point at the transition from the low-bias, high-temperature regime towards the high-bias, low-temperature regime (Figure SS2). Following Eq. 28, this transition occurs at the bias:

$$eV/(2k_\mathrm{B}T) \approx N_\mathrm{S}(\beta - 1). \tag{29}$$

As a result, the value of $eV/2k_\mathrm{B}T$ at the inflection point of the universal scaling curve provides a direct estimate of the apparent number of hopping steps N_S. As an example, this inflection point is indicated by the dotted line in panel (O) of Figure SS2. In Table S1, the resulting values of $N_\mathrm{S,USC}$ are listed for each of the 16 segments investigated. The corresponding estimate for the center-to-center distance $\delta_\mathrm{USC} = L/N_\mathrm{S,USC}$ ranges between 33 and 102 nm.

In the second approach, the value for N_S is numerically calculated from other parameters that are obtained by fitting the conductance data. To this end, we first note that for low temperatures and high voltages ($eV/N_\mathrm{S} \gg k_\mathrm{B}T$), the conductance scales as $G = B_0 V^\beta$, where B_0 is the pre-factor in the power law fit. Combining Eq. 18 and Eq. 26, this pre-factor can be explicitly written as:

$$B_0 = \frac{N_P}{N_S}\frac{\pi}{2\hbar}\left(\frac{e}{\hbar\omega_\mathrm{c}}\right)^{\beta+2}\frac{H^2}{\gamma(\beta+2)}\left(\frac{1}{N_S}\right)^{\beta} \tag{30}$$

Rearrangement provides an expression for the number of hopping sites:

$$(N_\mathrm{S})^{\beta+1} = \frac{N_\mathrm{P}}{B_0}\frac{\pi}{2\hbar}\left(\frac{e}{\hbar\omega_\mathrm{c}}\right)^{\beta+2}\frac{H^2}{\gamma(\beta+2)}. \tag{31}$$

Evaluation of this expression requires an estimate for the electronic coupling H. To arrive at this, we note that the pre-factor g_0 (as derived from the fitting of the Marcus rate expression, Eq. 10)

can be transformed into an expression for the electronic coupling:

$$H^2 = g_0 \frac{2\hbar}{\pi e^2} \frac{N_\mathrm{S}}{N_\mathrm{P}} \sqrt{4\pi\lambda}. \tag{32}$$

Substitution of Eq. 32 into Eq. 31 gives:

$$N_\mathrm{S} = \frac{e}{\hbar\omega_c} \left[\frac{\sqrt{4\pi\lambda}}{\gamma(\beta+2)(\hbar\omega_\mathrm{c})^2} \frac{g_0}{B_0} \right]^{1/\beta}. \tag{33}$$

From this, N_S can be calculated provided that we supply parameters that are fitted in the low-temperature regime (the pre-factor B_0, the coupling exponent β, the characteristic frequency $\hbar\omega_\mathrm{c}$) as well as parameters that are fitted in the high-temperature regime (pre-factor g_0, reorganization energy λ) via the Marcus rate expression Eq. 10.

The resulting values of $N_\mathrm{S,NUM}$ are listed in Table S2 for all 16 segments investigated. The associated center-to-center distance $\delta_\mathrm{NUM} = L/N_\mathrm{S,NUM}$ ranges between 10 and 36 nm, which is in good agreement with the corresponding estimate based on the universal scaling curve. Figure SS6 shows that the number of hopping sites linearly scales with the segment length. This trend is seen for both approaches.

References

1. van der Veen, J. R.; Valianti, S.; van der Zant, H. S.; Blanter, Y. M.; Meysman, F. J. A model analysis of centimeter-long electron transport in cable bacteria. *Physical Chemistry Chemical Physics* **2024**, 3139–3151.

2. Cornelissen, R.; Bøggild, A.; Thiruvallur Eachambadi, R.; Koning, R. I.; Kremer, A.; Hidalgo-Martinez, S.; Zetsche, E.-M.; Damgaard, L. R.; Bonné, R.; Drijkoningen, J.; others The cell envelope structure of cable bacteria. *Frontiers in Microbiology* **2018**, *9*, 3044.

3. Boschker, H. T.; Cook, P. L.; Polerecky, L.; Eachambadi, R. T.; Lozano, H.; Hidalgo-

Martinez, S.; Khalenkow, D.; Spampinato, V.; Claes, N.; Kundu, P.; others Efficient long-range conduction in cable bacteria through nickel protein wires. *Nature Communications* **2021**, *12*, 1–12.

4. Egger, R.; Mak, C.; Weiss, U. Quantum rates for nonadiabatic electron transfer. *The Journal of chemical physics* **1994**, *100*, 2651–2660.

5. Nazarov, Y. V.; Blanter, Y. M. *Quantum transport: introduction to nanoscience*; Cambridge University Press, 2009.

6. Jortner, J. Temperature dependent activation energy for electron transfer between biological molecules. *Journal of Chemical Physics* **1976**, *64*, 4860–4867.

7. Taylor, N. B.; Kassal, I. Generalised Marcus theory for multi-molecular delocalised charge transfer. *Chemical Science* **2018**, *9*, 2942–2951.

8. Wang, F.; Gu, Y.; O'Brien, J. P.; Sophia, M. Y.; Yalcin, S. E.; Srikanth, V.; Shen, C.; Vu, D.; Ing, N. L.; Hochbaum, A. I.; others Structure of microbial nanowires reveals stacked hemes that transport electrons over micrometers. *Cell* **2019**, *177*, 361–369.

9. Giannini, S.; Carof, A.; Ellis, M.; Yang, H.; Ziogos, O. G.; Ghosh, S.; Blumberger, J. Quantum localization and delocalization of charge carriers in organic semiconducting crystals. *Nature Communications* **2019**, *10*, 1–12.

10. Marcus, R. A.; Sutin, N. Electron transfers in chemistry and biology. *Biochimica et Biophysica Acta* **1985**, *811*, 265–322.

11. Kell, A.; Feng, X.; Reppert, M.; Jankowiak, R. On the shape of the phonon spectral density in photosynthetic complexes. *The journal of physical chemistry B* **2013**, *117 24*, 7317–23.
